# Lure, retain, and catch malaria mosquitoes. How heat and humidity improve odour-baited trap performance

**DOI:** 10.1186/s12936-020-03403-5

**Published:** 2020-10-07

**Authors:** Antoine Cribellier, Jeroen Spitzen, Henry Fairbairn, Cedric van de Geer, Johan L. van Leeuwen, Florian T. Muijres

**Affiliations:** 1 Experimental Zoology Group, Wageningen University, Wageningen, The Netherlands; 2 Laboratory of Entomology, Wageningen University, Wageningen, The Netherlands; 3 Faculty of Industrial Design Engineering, Delft University of Technology, Delft, The Netherlands; 4 Ifakara Health Institute, Ifakara, Tanzania

## Abstract

**Background:**

When seeking a human for a blood meal, mosquitoes use several cues to detect and find their hosts. From this knowledge, counter-flow odour-baited traps have been developed that use a combination of CO_2_, human-mimicking odour, visual cues and circulating airflow to attract and capture mosquitoes. Initially developed for monitoring, these traps are now also being considered as promising vector control tools. The traps are attractive to host-seeking mosquitoes, but their capture efficiency is low. It has been hypothesized that the lack of short-range host cues, such as heat and increased local humidity, often prevent mosquitoes from getting close enough to get caught; this lack might even trigger avoidance manoeuvres near the capture region.

**Methods:**

This study investigated how close-range host cues affect the flight behaviour of *Anopheles* female malaria mosquitoes around odour-baited traps, and how this affects trap capture performance. For this, a novel counter-flow odour-baited trap was developed, the M-Tego. In addition to the usual CO_2_ and odour-blend, this trap can provide the short-range host cues, heat and humidity. Systematically adding or removing these two cues tested how this affected the trap capture percentages and flight behaviour. First, capture percentages of the M-Tego with and without short-range host cues to the BG-Suna trap were compared, in both laboratory and semi-field testing. Then, machine-vision techniques were used to track the three-dimensional flight movements of mosquitoes around the M-Tego.

**Results:**

With heat and humidity present, the M-Tego captured significantly more mosquitoes as capture percentages almost doubled. Comparing the flight behaviour around the M-Tego with variable close-range host cues showed that when these cues were present, flying mosquitoes were more attracted to the trap and spent more time there. In addition, the M-Tego was found to have a better capture mechanism than the BG-Suna, most likely because it does not elicit previously observed upward avoiding manoeuvres.

**Conclusions:**

Results suggest that adding heat and humidity to an odour-baited trap lures more mosquitoes close to the trap and retains them there longer, resulting in higher capture performance. These findings support the development of control tools for fighting mosquito-borne diseases such as malaria.

## Background

Anthropophilic mosquitoes are vectors of dangerous diseases such as dengue fever and malaria, hence their host-seeking behaviour has been studied thoroughly [1]. Mosquitoes rely on the detection of CO_2_ and volatile odours to find human hosts [2, 3]. Then, like many other insects, mosquitoes perform a so-called cast and surge strategy to fly towards the odour source [2, 4–6]; they surge upwind when detecting an odour plume and cast crosswinds if they lose the plume. Finally, mosquitoes inspect visually contrasting objects and initialize landing in the presence of short-range host cues, such as heat or increased local humidity [3, 7–13].

Based on this knowledge on mosquito host-seeking behaviour, odour-baited traps have been developed [14–16]. These traps usually combine CO_2_, a bait mimicking human skin odours, and visual contrasts to attract mosquitoes [15, 17]. Most odour-baited traps have a single fan to generate a counter flow to dissipate the odour away from the trap, and capture mosquitoes by sucking them into the trap [15, 16, 18]. Odour-baited traps can attract many mosquito species and have been used successfully for years as research tools for monitoring mosquito populations [15, 19]. Recently, these traps have been considered as tools for integrated vector management, and used effectively in the field as such [20]. In the context of the recent worldwide slowdown of decrease in malaria cases, which is thought to be partly induced by the increasing mosquito resistance against widely used insecticides [1], such novel insecticide-free vector control tools are a promising alternative.

Understanding capture mechanisms of traps and flight dynamics around these traps is essential for further improvement of capture performance of the system. But only a few studies looked at mosquito flight behaviour in the vicinity of odour-baited traps. First, Cooperband and Cardé analysed three-dimensional flight tracks of two *Culex* mosquito species flying towards CO_2_-baited traps in a semi-field tent [21]. They showed that differences in capture percentages between the traps correlated with changes in the observed flight dynamics of mosquitoes.

More recently, Cribellier et al. reconstructed thousands of three-dimensional flight tracks of malaria mosquitoes (*Anopheles coluzzii*) interacting with the BG-Suna trap (Biogents, Germany) in its default hanging position and in an opposite standing orientation [18]. The standing orientation made the studied BG-Suna analogous to a BG-Sentinel trap (Biogents, Germany), which has been developed for capturing *Aedes* mosquitoes. The standing BG-Suna was found to be more attractive and overall performed better than the hanging BG-Suna. However, only the standing BG-Suna elicited upward avoiding manoeuvres from mosquitoes flying above its inlet. Such avoiding behaviour may be due to the lack of short-range host cues or be mediated by the high-speed suction flow generated by the traps [18, 22]. As a result, only a low percentage of flight trajectories near the trap led to capture in both trap orientations [18]. Despite the fact that the BG-Suna trap seems to have a relatively low capture efficiency, long-term employment in the field resulted in significant reductions in mosquito populations and a decrease in malaria incidence [20]. Follow-up studies confirmed these findings on mosquito flight dynamics around traps. Batista et al. found that *Anopheles arabiensis* follow similar flight patterns below the BG-Malaria trap [23]. This trap is adapted from an upside-down BG-Sentinel to trap anopheline mosquitoes. Finally, by studying the flight behaviour of *Aedes aegypti* around the BG-Sentinel, Amos et al. also observed upward avoidance responses near the trap inlet, and confirmed that tested traps showed low capture efficiency [22].

Several mosquito traps use short-range host cues such as heat and humidity to increase capture performances [21, 24]. Odour-baited CDC-type traps (Centers for Disease Control, USA) were found to capture significantly higher numbers of *Aedes*, *Anopheles* and *Culex* mosquitoes when generating heat using a 50 W moist heating pad [25]. Mosquito Magnet traps run on propane gas that is catalytically converted to produce CO_2_, heat, and moisture [21]. Heat was also found to be a crucial cue to elicit the landings of anopheline mosquitoes necessary for the Human Decoy Trap (HDT) [9, 17, 26]. This adhesive trap uses a combination of CO_2_ and odour provided from people or cattle in a nearby tent, with visual cues and heat generated by a black container filled with warm water (surface temperature of 35 ± 5 °C) [17, 27]. Despite their good capture performance, these traps have a number of practical drawbacks for potential use as vector control tools against *Anopheles* mosquitoes. The CDC-type traps with a heating pad require high electric power input for generating heat. The Mosquito Magnet traps are expensive and distribution to malaria-endemic countries would be more than challenging. The HDT trap requires live bait as well as the warming up of a large quantity of water at high temperature (~ 80 °C) [27].

It is still unknown how adding heat and increased local humidity to odour-baited traps affects mosquito flight behaviour near the trap entrance. This study tested the hypothesis that without close-range host cues such as heat and humidity, mosquitoes often do not get close enough to odour-baited traps to get caught, resulting in a low capture efficiency. This was done by studying the flight behaviour of *Anopheles* around a new counter-flow odour-baited trap, the M-Tego. This trap was developed by the authors in order to be able to generate both heat and humidity in addition to the CO_2_, odour blend and visual cues that are usually implemented in odour-baited traps. Additionally, the M-Tego is easy to transport and maintain and as such, has the potential for a wide use in vector control programmes [20]. As a benchmark, the capture percentages of the M-Tego was compared to the ones of the standing BG-Suna trap in both laboratory and semi-field conditions. The M-Tego without or with additional cues was found to capture significantly more mosquitoes than the standing BG-Suna in the laboratory and in semi-field conditions.

To investigate why such high capture percentages were observed, the flight behaviour of mosquitoes around the M-Tego was recorded, with or without short-range host cues. Despite high similarities between flight behaviour around the BG-Suna and the M-Tego, it was found that, if additional host cues were present, flight activity greatly increased in a region around the rim of the odour outlet. Finally, contrary to what has been found for the BG-Suna, avoidance behaviours of mosquitoes were not observed above the M-Tego. This lack of avoidance manoeuvres may explain why the M-Tego even without additional host cues had higher capture percentages than the BG-Suna. These results are promising for integrated vector control programs to improve human health and welfare.

## Methods

### Experimental animals

For all laboratory experiments at Wageningen University, female *Anopheles coluzzii* mosquitoes were used. These came from a laboratory-reared colony that originated from Suakoko, Liberia in 1987. The colony is housed in the Laboratory of Entomology (Wageningen University & Research, The Netherlands) with a clock shifted 12 h light:12 h dark cycle, and at fixed temperature of 27 °C and relative humidity of 70%. Adults were kept in BugDorm (MegaView Science Co. Ltd., Taiwan) cages (30 × 30 × 30 cm) and fed sugar water solution with 6% glucose. Additionally, they were blood-fed daily with human blood (Sanquin, Nijmegen, The Netherlands) using a membrane feeding system (Hemotek, Discovery Workshop, UK). Female mosquitoes could lay their eggs on wet filter papers that were then moved to plastic trays filled with water. Emerging larvae were fed with Liquifry No. 1 fish food and TetraMin Baby (Tetra Ltd, UK). Finally, new pupae were placed in new BugDorm cages to emerge. Males and females were kept together so they could mate. Non-blood-fed adult females (age = 9.8 ± 1.4 days (mean ± std)) were collected between 12 and 16 h before experiments.

Semi-field experiments in Tanzania were done using 3 to 8 days old *Anopheles gambiae s.s.* female mosquitoes. These mosquitoes were reared under standard insectary conditions of 27 ± 5  °C (room temperature), 40–100% relative humidity and a 12L:12D cycle. Larvae were fed ad libitum on TetraMin fish flakes (Tetra Ltd., UK). Adult mosquitoes were kept in metal cages (30 × 30 × 30 cm) and fed ad libitum on a 10% glucose solution. Female mosquitoes used for the rearing were blood-fed with cow blood using a membrane feeding system (Hemotek).

### Odour-baited traps

The traps used in these experiments were the BG-Suna (Biogents, Germany) and prototypes of the new M-Tego trap (PreMal b.v., The Netherlands). The BG-Suna was used in a standing orientation, thus mimicking the BG-Sentinel, as this position was found to have a better capture efficiency and attractiveness than the hanging BG-Suna [18, 28]. In both traps, an odour source containing one MB5 blend was used to simulate human skin odour [28, 29], and CO_2_ to simulate human breath. For the laboratory experiments, CO_2_ was provided using a pressurized canister, and consisted of a mixture of 5% CO_2_ with 95% air at a flow rate of 200 ml/min. For the semi-field tests, CO_2_ was produced using a mixture of 17.5 g of yeast and 500 g of molasses in 2 L of water [30, 31]. As in Cribellier et al. [18] the CO_2_ pipe of the BG-Suna was shortened and the top of the inlet was levelled to minimize blind spots of the cameras.

The M-Tego is a novel trap developed by the authors (see author contributions for details) at Wageningen University (The Netherlands) in collaboration with industrial designers from Delft University of Technology (The Netherlands) (see Additional file 1: Fig. S1). In this study, prototypes of this novel trap were used, from here on those prototypes will be referred to as M-Tego traps. The M-Tego trap uses a similar counter-flow principle as the BG-Suna to attract and capture mosquitoes, and both traps use the same brushless 12 V dc fan. With a diameter of 30 cm and a height of 38.8 cm, the M-Tego is smaller than the BG-Suna trap that has a diameter of 52 cm and a total height of 39 cm. Its inlet is slightly higher than that of the BG-Suna (9.5 cm vs 8.3 cm with levelled inlet) but both inlets have the same diameter of 11 cm. The M-Tego has a foldable black polyester tarpaulin bag (70 g per sq m, Gamma, The Netherlands), which makes transportation easier, as well as an HDPE insect net (Howitec, The Netherlands) on the top of the tarpaulin bag, to allow the outward circulation of the odour-saturated air. Additionally, the M-Tego uses an inlet module with an integrated catching cage that simplifies the removal of caught mosquitoes (see Additional file 1: Fig. S1). These design decisions improve user-friendliness and aim to reduce fabrication costs, which is beneficial for a vector control tool in rural Africa. To generate heat similar to that produced by a human body (37 °C), the M-Tego uses a 2-m Nichrome wire (diameter 0.5 mm) wrapped around the top of its inlet. The heater has an electric power requirement of 9.6 W (12 V and 0.8 A)). Finally, the trap can be filled with 1 L of warm water at 40 °C to increase local relative humidity and temperature. The wire is not in contact with the water and thus cannot warm it up. Instead, the water needs to be warmed up passively during the day or using exterior means.

### Experimental set-ups

Three experiments were performed. First, in dual choice testing in the laboratory, two traps were placed next to each other in a flight tent. Mosquitoes were released in the tent where they were free to fly around the two traps. Using this set-up, trap capture percentages were compared to each other. Secondly, semi-field experiments were performed inside three large screen houses in Tanzania. In each screen house, one of the tested traps was placed next to a replicate of a rural African house. The numbers of released mosquitoes that were captured by each trap were then compared. Third, to study the flight behaviour of mosquitoes around the M-Tego, with or without additional host cues, their flight trajectories were tracked in the laboratory in the vicinity of the trap using machine vision techniques.

### Dual-choice experiments

**Fig. 1:**
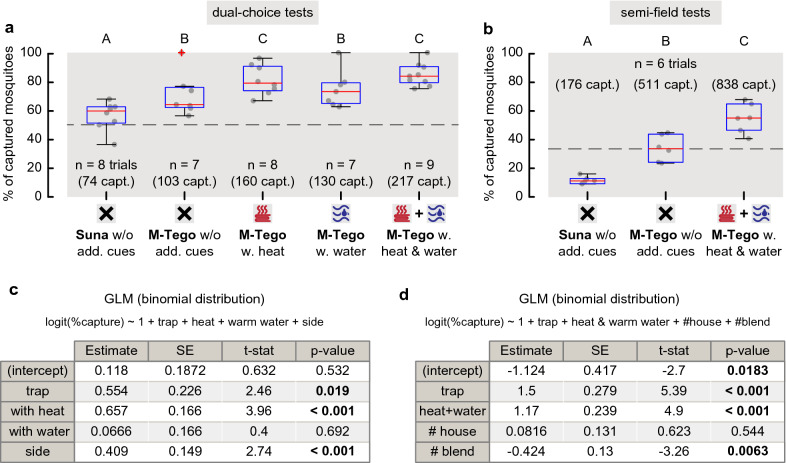
Comparing capture performance of the BG-Suna and the M-Tego, with and without close-range host cues. Capture percentage of the BG-Suna and the M-Tego with or without additional host cues against a second BG-Suna in dual-choice tests (**a**) and in separated screen houses in semi-field tests (**b**). Dashed line shows expected percentage if no differences existed between the traps. Letters above box plots indicate results that did not differ significant (GLM, p > 0.05). Total numbers of captures per condition are indicated in parentheses. **c**, **d** Formula and results of the minimal GLM used to model variation of capture percentages in **a** and **b**, respectively

The goals of the dual-choice tests were, first, to benchmark the capture performance of the M-Tego by comparing it to the well-established BG-Suna and, secondly, to quantify the effect of adding short-range host cues on the capture performance of the M-Tego. Five trap conditions were tested versus the same BG-Suna: the BG-Suna #2 (control), the M-Tego without additional cues, the M-Tego with heat, the M-Tego with warm water, and the M-Tego with heat and warm water (Fig. 1a).

The dual-choice tests were performed in a netting cage of 2.9 × 2.5 × 2.5 m (Howitech, The Netherlands, see Additional file 1: Fig. S2) inside a climate-controlled room (ambient temperature = 26.1 ± 0.9 °C (mean ± std), and relative humidity = 72.9% ± 3.9%). On each side of the cage, a trap could be placed above the centre of a 1 × 1 m horizontal white ground plate. These two plates were placed in front of two 1 × 2 m vertical white plates and next to each other, separated by another 1 × 2 m vertical plate. All traps were placed in the cage such that the top of the trap inlet was at a height of 54.5 cm, in order to be consistent with our previous study [18]. During the experiments, the room was illuminated only by a single nightlight (0.4 W), placed above the centre of the cage.

Each trap was equipped with a MB5 odour source (OS1 or OS2) and placed on the left or right side of the cage. The position of the traps (left or right) and the odour source used (OS1 or OS2) were chosen following a quasi-randomized planning where all combinations of conditions were tested at least 7 times. See Additional file 2: Database S1 for a summary of all test conditions.

Before each experiment, the traps and the experimental set-up were cleaned using a 15% ethanol solution. All handling of the materials and mosquitoes was done wearing nitrile gloves to minimize the risk of skin odour contamination. After setting up the traps, 50 mosquitoes were released from a holding container on the opposite side inside the netting cage by pulling a string outside of the cage. Then, the experimenter left the room. Mosquitoes could then choose to fly around their preferred trap. After 20 min, the experimenter re-entered the room, closed the traps, killed the remaining mosquitoes in the cage using an electric mosquito zapper and cleaned the cage with a vacuum cleaner. Each trap capture bag was then placed in a freezer to kill captured mosquitoes, which were manually counted later. Relative humidity and temperature inside the room were recorded before and after each trial using a weather station (TFA Dostmann/Wertheim, Kat. Nr. 30.5015). Four such dual-choice trials were done during each experimental morning, which coincided with the dark period of night-active mosquitoes.

### Semi-field testing

To verify the results of the dual-choice laboratory tests, the capture percentage of the BG-Suna and the M-Tego with or without heat and warm water in semi-field experiments were compared. These experiments were performed at the Ifakara Health Institute (Ifakara, Tanzania) during the first week of November 2018. For the experiments, three screen houses of 10 × 10 m each were used with a slightly scaled-down house inside (see Additional file 1: Fig. S3). These houses were built from local materials such as bricks, corrugated sheet metal, straw or mud. Three traps were tested each experimental night, a BG-Suna, a M-Tego without additional host cues and an M-Tego with heat and warm water (Fig. 1b). The tested traps were placed outside the houses, with their inlet pipe at a height of 65 cm. The fact that this height is higher than the one in the laboratory experiments (65 vs. 54.5 cm) might have resulted in a small overall difference in capture performances [15]. However, it is unlikely that it would have affected the comparative results between traps. The M-Tego traps were hung from the house, whilst the BG-Suna was placed in a metal wire frame on the ground. The traps were cleaned before use with 70% ethanol and handled with gloves afterwards. Each trap was powered using a 12 V car battery and contained an MB5 odour source. CO_2_ was produced using 5.5 L plastic containers with a yeast and molasses mixture, which was placed next to each trap, and replaced daily. Inside each house, a set-up with CO_2_, an MB5 blend and a fan (same as used in the traps) was placed below a bed net to simulate human presence. The fan was positioned such that it produced an airflow that directed the odour and CO_2_ towards the nearest window. Screen houses were cleaned before and after the experiments. Only natural (moon) light was illuminating the screen house during the night.

Before each trial, each trap was equipped with one of three MB5 odour sources (OS3, OS4 or OS5), and placed inside one of the three screen houses. The odour source and the screen house used for each trap were changed following a quasi-randomized planning (see Additional file 2: Database S2). The M-Tego with short-range host cues was then filled with 0.7 L of water from a water bottle that stood in direct sunlight during the day (temperature = 39.7 ± 0.5 °C, n = 2). At the start of the experiment, a release pot containing 200 females *An. gambiae s.s.* was placed in the corner of each screen house and the mosquitoes were released manually, at approximately 18:00 h. The experiment ended around 06:20 the following morning. Captured mosquitoes were killed by moving the capture bag or pot out of the trap and placing them in direct sunlight for a day. The desiccated mosquitoes were manually counted.

The mean run-time of the experiments was 12 h and 23 min (± 22 min). At the start of the experiment, the ambient air temperature was 32.4 ± 1.4 °C and ambient relative humidity of the air was 38.1 ± 4.7%. The next morning, the ambient air temperature was 22.9 ± 0.8 °C and ambient relative humidity was 78.0 ± 6.0%.

### Mosquito flight tracking experiments

To study the flight behaviour of mosquitoes around the M-Tego with or without additional host cues, mosquitoes were tracked around the traps in the same netted cage as used for the dual-choice tests (ambient temperature = 24.9 ± 0.7 °C and relative humidity = 73.7 ± 3%). The experimental procedure was identical to the dual-choice experiments, except for the following differences. A single M-Tego was placed on the right side of the dual-choice setup and a single MB5 odour source was used inside the trap (OS1). Four trap conditions were tested, the M-Tego without additional cues, the M-Tego with heat, the M-Tego with warm water, and the M-Tego with heat and warm water. Each experimental morning, all conditions were tested using as quasi-randomized planning (see Additional file 2: Database S1).

**Fig. 2:**
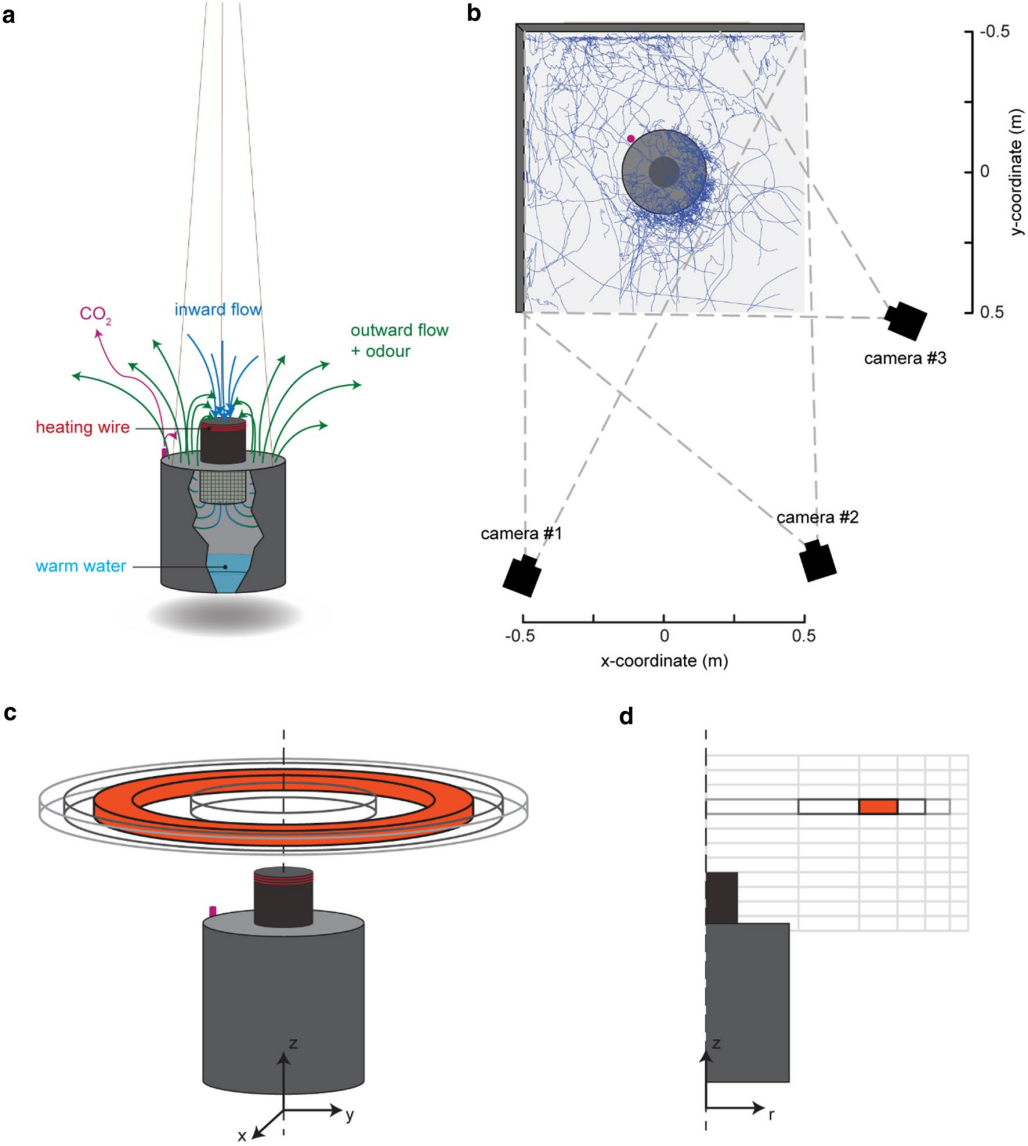
Experimental setup of the mosquito-tracking experiments, and analysis method for visualizing the three-dimensional flight dynamics in two-dimensional heat maps. **a** Schematic of the M-Tego with a removed slice to make the inside visible. The fan inside the inlet generates a circulating airflow by sucking air inside (blue arrows), mixing it with the odour blend and CO_2_, and then pushing air away from the trap (green arrows). Additionally, the trap can be filled with 1 L of warm water and heat can be generated by a Nichrome heating wire at the top of the inlet. **b** Top-down view of the experimental setting used for recording mosquito flight behaviour around the M-Tego. The filmed region is delimited by the angles of view of the 3 cameras (dashed grey lines). Flight tracks recorded during one trial are visualized in blue. Method for projecting three-dimensional rings around the trap (**c**) into two-dimensional surfaces (**d**). **c** The filmed volume was divided in three-dimensional rings of equal volume and centred around the trap axis of symmetry. **d** Each ring is projected into a cell in a two-dimensional parametric space comprising the vertical position (z-coordinate) and the radial distance to the trap’s axis of symmetry. In this way, heat maps of various metrics have been computed to visualize flight behaviour

Three infrared-enhanced cameras (Basler acA2040-90umNI) with Kowa 12.5 mm lenses (LM12HC f1.4) were used for the tracking (Fig. 2), which synchronously recorded images at temporal resolution of 90 frames per second and a spatial resolution of 1024 × 1024 pixels. Cameras were synchronized using pulses from an Arduino Uno board. Because mosquitoes cannot see infrared light [32], the tracking set-up was illuminated using two infrared light-emitting-diode (LED) lamps (Bosch Aegis SuperLed, 850 nm, 10° beam pattern – SLED10-8BD). Lens distortions were corrected using pictures of a chequerboard pattern. Calibration was done daily using a single LED manually waved inside the filmed volume to find DLT (direct linear transformation) coefficients [33]. Alignment was done with a calibration device with four LEDs that were consecutively blinking at various known three-dimensional positions. The real-time three-dimensional tracking software Flydra (version 0.20.19) was used to track the three-dimensional positions, velocities and accelerations of flying mosquitoes within a three-dimensional space of approximately 1x1x1 m around the trap [34]. For each experiment, mosquito flight trajectories were recorded for 20 min, but the first 3 min were used for tracker initialization. The remaining 17 min were used for the analysis.

### Analysis of three-dimensional flight tracks

All the flight dynamics analyses were done using Matlab 2018b (MathWorks). Mosquitoes could enter and exit the filmed three-dimensional space several times per trial, therefore individuals could not be identified. Filtering of tracked points was based on the covariance matrices estimated by the extended Kalman filter used for three-dimensional reconstruction by Flydra. Three-dimensional points with too high estimated standard deviation of either their position or speed were considered as outliers and deleted. When two or fewer consecutive video frames had missing values in the three-dimensional tracks, they were linearly interpolated. If more than two consecutive frames had missing values, the trajectory was divided in two separate tracks. Finally, tracks shorter than 10 video frames in length were deleted. For all frames of each computed three-dimensional track, linear and angular flight speeds as well as linear accelerations were computed (as in  [18]).

To systematically analyse the flight behaviour of mosquitoes around the M-Tego trap, the visualization technique developed by [18] was used: based on the verified assumption that the three-dimensional flight behaviour is on average axially symmetric around the trap axis of symmetry, the three-dimensional flight movements can be projected onto a two-dimensional sub-space [18]. For this, the three-dimensional space was divided into multiple three-dimensional rings centred around the M-Tego axis of symmetry (Fig. 2a, c). Within each ring, all relevant flight dynamics metrics (such as mean flight speed) were computed. Results were visualized by projecting the metric value in each ring onto a two-dimensional parametric space with the radial and vertical positions as coordinates (Fig. 2d). All rings had a constant volume to allow metric comparison. In addition, all metric results were visualized using two-dimensional heat maps from a top-down view and from a side view close to the background walls (see Additional file 1: Figs. S5, S7, S9 and S12). For these the three-dimensional space around the trap was divided into vertical and horizontal columns, respectively.

**Fig. 3:**
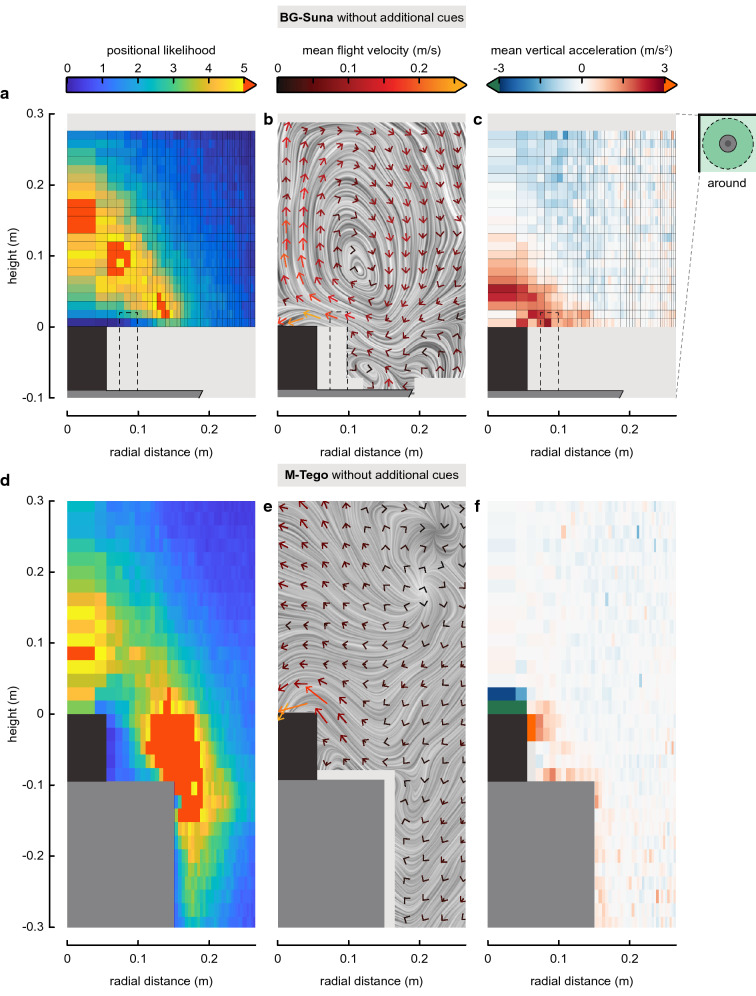
Comparison of the flight dynamics of mosquitoes around the BG-Suna and the M-Tego. Heat maps of the positional likelihood of mosquitoes flying around **a** the BG-Suna (from Cribellier et al. 2018) and **d** the M-Tego without additional host cues. The positional likelihood *P*_*i*_ in a cell *i* is defined as the normalized probability of mosquitoes to fly inside a given three-dimensional ring around the trap (average cell size = 19 × 3 mm). Random flight behaviour would result in a uniform probability equal to 1 throughout the filmed volume. Average velocity fields and streamlines of mosquitoes flying around **b** the BG-Suna and **e** the M-Tego without additional host cues. Each vector consists of the average velocity in the radial and vertical direction of all mosquitoes that flew in a cell (size = 27.5 × 27.5 mm). All velocity vectors resulting from fewer than 20 tracks were discarded. Streamlines are shown using line integral convolution (LIC). Heat maps of the average vertical acceleration of mosquitoes flying around **c** the BG-Suna, and **f** the M-Tego without additional cues

**Fig. 4:**
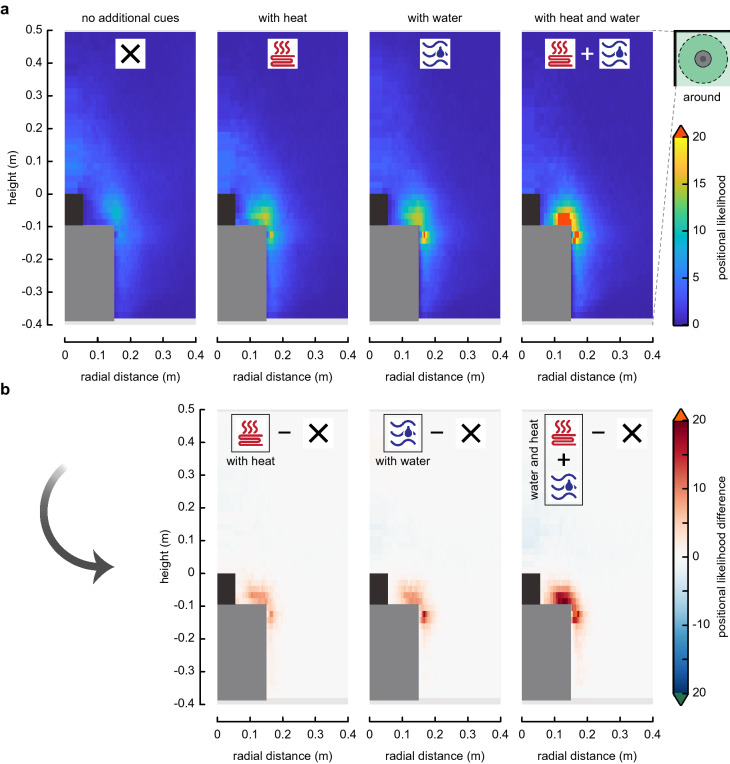
Spatial distribution of the positional likelihood of mosquitoes flying around the M-Tego with and without additional cues. **a** Radial–vertical heat maps of the positional likelihood around the M-Tego with various combinations of additional host cues. The positional likelihood *P*_*i*_ in a cell *i* is defined as the normalized probability of mosquitoes to fly inside a given three-dimensional ring around the trap. Random flight behaviour would result in a uniform probability equal to 1 throughout the filmed volume. Average cell size = 19 × 3 mm. **b** Difference between the heat maps of positional likelihood around the M-Tego with various additional host cues (heat and/or water) and the heat maps of positional likelihood around the M-Tego without additional host cues

For each tested condition, the following metrics were computed in all cells (rings or columns) around the trap: the positional likelihood of mosquitoes in each cell (Figs. 3a, d, 4a), the average time spent in a cell, the average flight velocity, flight speed, upward acceleration, angular speed, and capture probability.

The capture probability of a mosquito flying in a specific cell is defined as the number of tracks in that cell that ended by a capture divided by the total number of tracks detected in the cell. The positional likelihood of mosquitoes in a cell is defined as the normalized probability of a mosquito to fly in a cell (i.e., a horizontal ring projected in the two-dimensional parametric space). This metric allows the identification of regions of high flight activity. The positional likelihood $P_{i}$ of mosquitoes to fly in cell *i*, was computed as $P_{i} = \frac{{n_{i} }}{N} \times I$, where $N$ is the sum of the length of all tracks in the filmed three-dimensional space (i.e. the total number of frames), $n_{i}$ is the sum of the length of the parts of these tracks with mosquitoes flying in cell *i*, and $I$ is the total number of cells within the three-dimensional space. In this way, if flight behaviour was random and the number of tracks was high, the positional likelihood heat-maps would be uniform with a value of 1. The average time spent by mosquitoes in a cell is defined as the total time spent by all mosquitoes in a cell divided by the number of tracks detected in that cell. Because the positional likelihood in a cell is equivalent to the normalized total time spent by all mosquitoes in a cell, the average time spend is a good metric to weight the measure of positional likelihood. For details about the other metrics, see [18].

To test how close-range cues affected the flight dynamics of mosquitoes, the difference in each metric between treatments were computed by subtracting the results distribution around the M-Tego without additional cues from the treatments with heat and/or warm water (Fig. 4).

### Statistical analysis

For all statistical tests, generalized linear models (GLM) were used. For each test, the minimal model was determined by successively removing the least significant predictors from the model until all remaining predictors were significant (*p* value < 0.05). We then chose the GLMs with the lowest AIC (Akaike Information Criterion) values (see Additional file 2: Table S1).

For the dual choice experiments, a GLM with a binomial distribution, logit link function and estimated dispersion [35] was used. The response variable of the GLM was the capture percentage, defined as the ratio between number of captures by one trap and the number of captures by both traps. The predictors for the full model were: trap (BG-Suna or M-Tego), with or without heat, with or without warm water, mean humidity, mean temperature. Location (left or right side) and the odour source used in the trap (OS1 or OS2) were also included. When determining the minimal model, the predictors of interest “trap”, “heat” and “warm water” in the model were always kept.

For the semi-field experiment, a GLM with a binomial distribution, logit link function and estimated dispersion was also used. The response variable of the GLM was the capture percentage, defined as the ratio between number of captures of one trap and the total number of captures of all traps across all three screen houses. The full model predictors were trap (BG-Suna or M-Tego), with/without heat and warm water, mean humidity, mean temperature, day of the experiment, house number (H1, H2 or H3), and the odour source used in the trap (OS3, OS4 or OS5).

To compare the flight behaviour of the mosquitoes around the M-Tego trap with or without host cues, three doughnut-shaped volumes were defined around the top rims of the trap with various radii of their tube (r = $\left[ {\frac{1}{2}, \frac{3}{4}, 1} \right] \times \left( {r_{bag} - r_{inlet} } \right)$, with $r_{bag}$ and $r_{inlet}$ being, respectively, the radius of the tarpaulin bag and radius of the inlet of the trap). The positional likelihood of mosquitoes to be tracked inside these volumes as well as how long on average they stayed inside for each trial was then computed (Fig. 6). Because mosquitoes were not tracked during the dual-choice and semi-field experiments, these two metrics could not be used as covariate in the previously described models. GLMs with a gamma distribution, negative inverse link function and estimated dispersion were used to model how these two metrics varied as a function of the presence of heat and warm water. Full model predictors were with heat (yes or no), with warm water (yes or no), mean humidity, mean temperature, volume of the doughnut and age in days of the adult female mosquitoes.

## Results

### Capture efficiency of the traps

A total of 39 dual-choice trials were performed in the laboratory (Fig. 1a) and 907 female mosquitoes were caught by the traps, for a total of 1950 released mosquitoes (more details in Additional file 2: Database S1). Here, the capture performance of the M-Tego with various additional short-range host-cues was compared to that of the BG-Suna (Fig. 1). The M-Tego without additional short-range host-cues captured on average 70.8% ± 13.6% (mean ± std, 7 trials) of the total number of mosquitoes captured by the M-Tego and BG-Suna combined, which was significantly higher that the capture percentage of the BG-Suna in control experiments (GLM, p = 0.019). Adding heat to the M-Tego significantly increased this capture percentage to 81.1% ± 9.7% of all captured mosquitoes (GLM, p < 0.001, 8 trials). The M-Tego with only warm water captured 74.8% ± 12% (7 trials) of all captured mosquitoes, and the M-Tego with both warm water and heat captured 83.8% ± 7.3% (7 trials), but the effect of adding warm water to the M-Tego (with (9 trials) or without heat (7 trials)) was not significant (GLM, p = 0.692). On average the M-Tego without additional cues and with heat captured 2.4 and 4.3 as many mosquitoes than the competing BG-Suna.

A total of six tests were performed in semi-field conditions (Fig. 1b) in which the capture performance of the BG-Suna, the M-Tego without additional cues and the M-Tego with warm water and heat were compared. 3600 female mosquitoes were released, of which a total of 1525 were caught (see Additional file 2: Database S2 for details). The M-Tego without additional host cues captured on average 33.8% ± 8.3% of all mosquitoes captured by the three traps, the M-Tego with warm water and heat captured 54.7% ± 9.4% of these mosquitoes, and the BG-Suna captured only 11.5% ± 2.4% of all captured mosquitoes (Fig. 1b). The capture percentages of all three traps differed significantly from each other (GLM, p < 0.001, Fig. 1), showing that on average the M-Tego without and with additional cues captured 2.9 and 4.7 as many mosquitoes than the BG-Suna, respectively.

### Flight dynamics around the M-Tego

The flight dynamics of mosquitoes around the M-Tego trap were monitored, with or without additional host cues for a total of 18 h and 25 min (65 trials). During these trials, 3250 mosquitoes were released, 13,618 flight trajectories were reconstructed, and 1335 of these tracks led to capture. The number of tracks per trial that were identified to result in a capture was highly correlated with the number of mosquitoes caught inside the traps (1600 mosquitoes in total, correlation coefficient = 0.937). This suggests that the detection of tracks leading to capture was good.

**Fig. 5:**
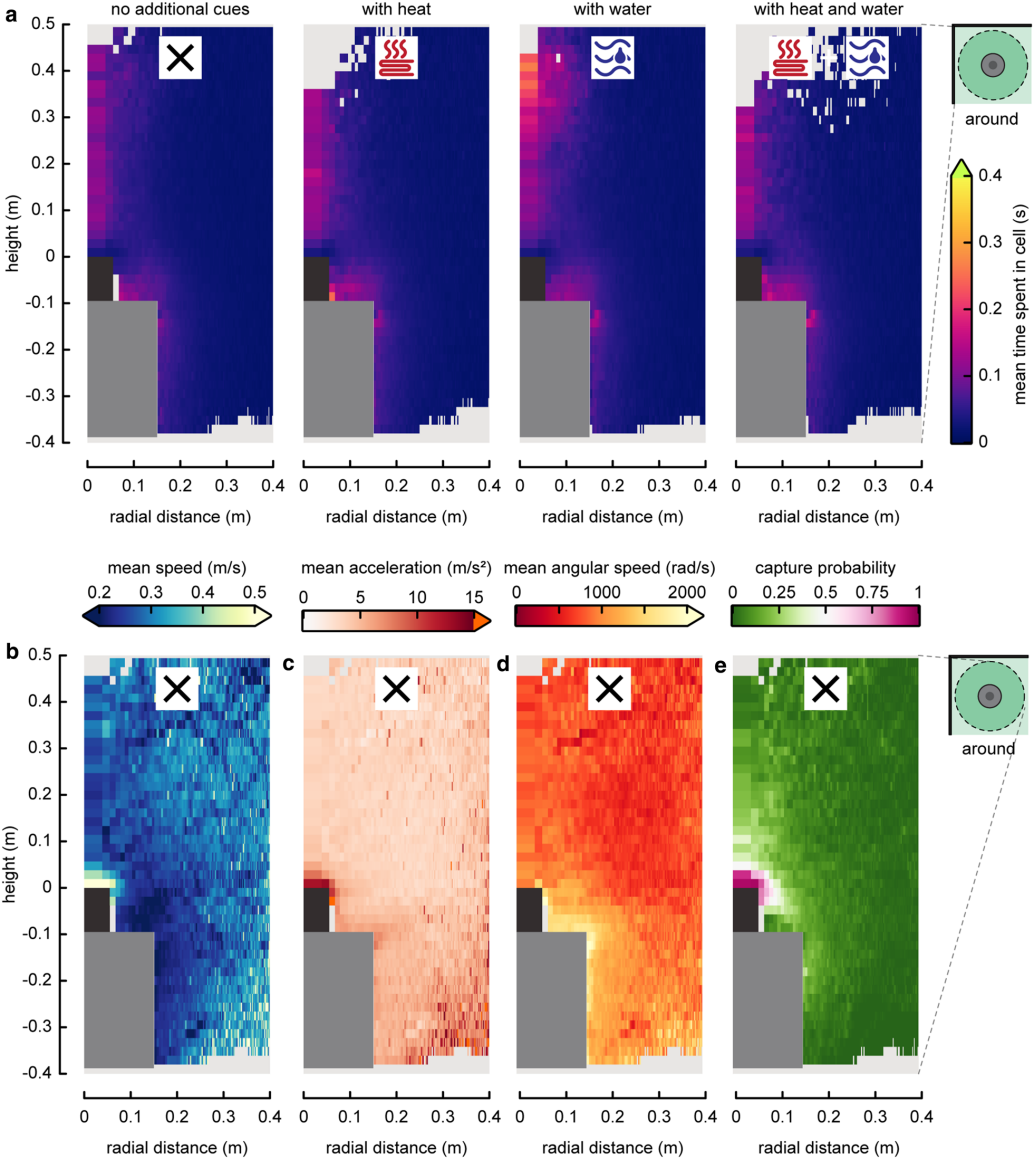
Heat maps of various flight dynamics metrics of mosquitoes flying around the M-Tego. **a** Heat maps of the average time spent by mosquitoes around the M-Tego with various combinations of additional host cues. **b**–**e** Heat maps of the average flight speed, average acceleration, average angular speed and capture probability of mosquitoes flying around the M-Tego (without additional cues). Cells with fewer than 20 tracks have been masked. Average cell size = 19 × 3 mm. See Additional file 1: Figures S10–S12 for additional heat-maps of these metrics

From all these tracks, various heat maps were computed to visualize the average flight behaviour of the mosquitoes around the traps (Figs. 3, 4, 5). Positional likelihood around the M-Tego without additional cues was similar to the ones observed above the BG-Suna [18]. Near both traps, mosquitoes were likely to be found flying in a cone-shaped region close to the traps (Fig. 3). Their positional likelihood was especially high close to the rim of the top surface where odour is released. Despite this similarity in positional likelihood, the average flight dynamics around the BG-Suna and the M-Tego traps exhibited clear differences. Although mosquitoes had similar flight paths towards the inlets of both traps, only mosquitoes flying above the BG-Suna exhibited an upward-directed avoiding behaviour (Fig. 3b, e). This difference is highlighted by the heat maps representing mean vertical acceleration (Fig. 3c, f). Indeed, mosquitoes close to the M-Tego inlet were on average only accelerating downward (i.e., when being caught), while above the BG-Suna mosquitoes were also found to avoid capture by accelerating upwards [18]. Flight paths in other regions near the M-Tego do not show any clear directional patterns (Additional file 1: Fig. S7).

Two distinct behavioural regions can be defined based on the spatial distributions of the remaining computed flight metrics around the M-Tego (Fig. 5b–e). In the first region, just above and around the trap tarpaulin bag, mosquitoes had on average lower flight speeds and higher angular speeds (Fig. 5b, d) than in the rest of the filmed volume. In the second region, above the trap inlet, mosquito flew faster and produced on average higher accelerations (Fig. 5b, c). In this last region, the capture probability was also the highest (Fig. 5e), meaning that most tracks detected there led to capture.

### Effect of heat and warm water on flight behaviour

**Fig. 6:**
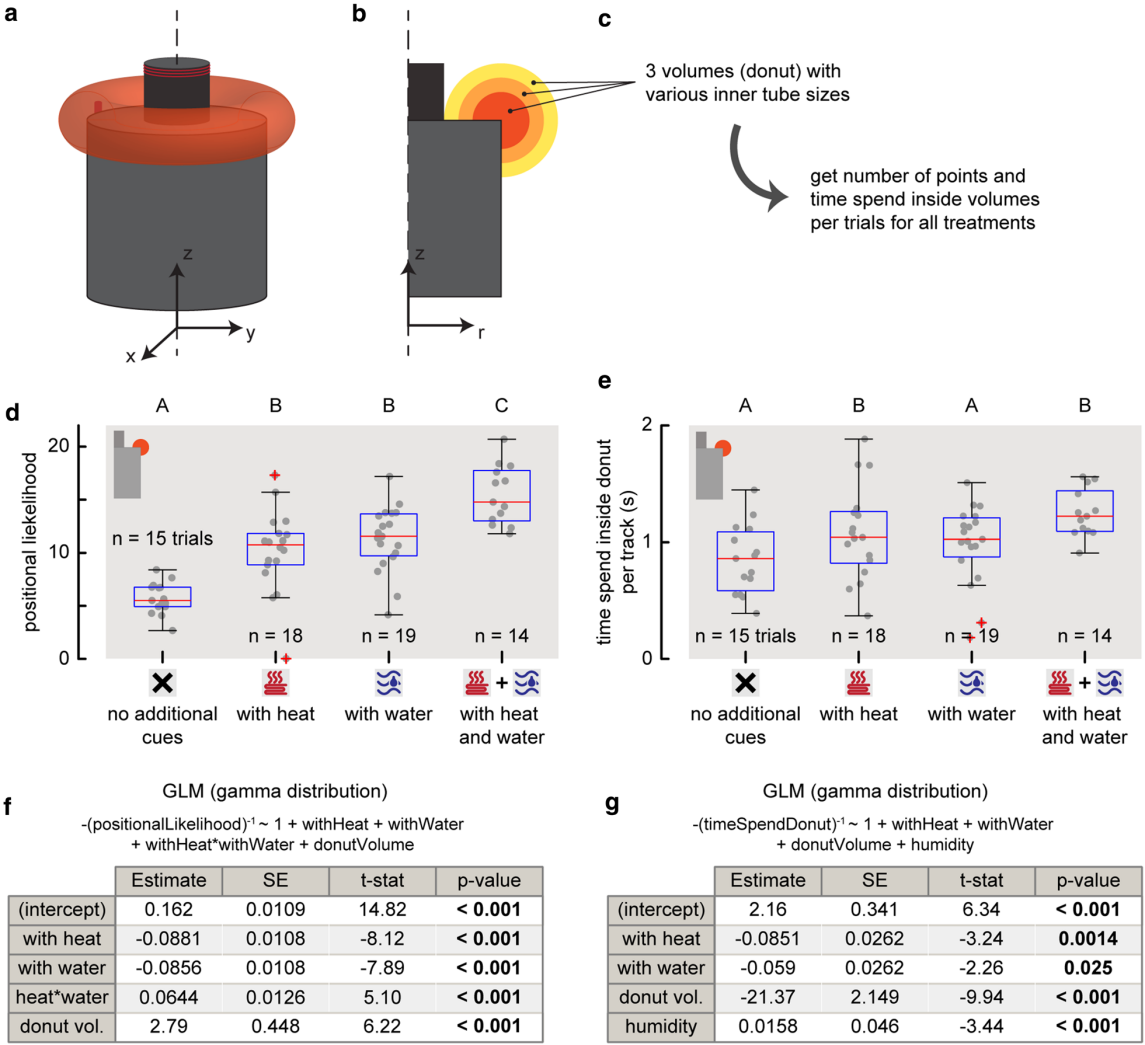
The positional likelihood and time spent in the donut-shaped region close to the trap edge, and how these differed for traps with variable additional host cues. Method description, showing **a** a three-dimensional view of the donut shaped volume used to compare flight behaviour around the trap, **b** a transverse section through three donuts with variable radii, and **c** the method explanation for the comparison between the different treatments. **d**–**g** Statistical results for the largest donut-shaped volume (yellow in **b**), using 7153 tracks. Results for the two smaller volumes are shown in Additional file 1: Figure S13. **d** Box plots of the positional likelihood inside the largest donut-shaped volume (yellow in **b**) of mosquitoes flying around the various traps with or without additional cues. Each datapoint shows the result of a single trial. **e** Box plot of the average time spend inside the largest volume (yellow in **b**) by mosquitoes flying around the different traps with various additional cues. **d**, **e** Each datapoint shows the result of a single trial, and different letters above each box plot indicate significance difference between treatment (GLM, p < 0.05). **f**, **g** Formula and results of the generalized linear mixed models (GLM) used to model the metrics from **d**, **e**, respectively

To compare the flight behaviour of mosquitoes around the M-Tego with or without additional host cues, heat maps of their positional likelihood (Fig. 4a) and time spent close to the traps (Fig. 5a) were computed. On these heat maps, a higher flight activity was observed close to the M-Tego when additional host cues were added. By adding heat to the M-Tego, the positional likelihood around the rim of the trap increased by 79.0% ± 49.5%; when adding warm water, the positional likelihood increased by 98.3% ± 52.3%; when adding both heat and warm water, the positional likelihood around the trap rim was increased by 171.1% ± 79.5% (GLM with p < 0.001, Fig. 6d, f). Additionally, mosquitoes have been found to fly on average 21.2% ± 38.5% longer in this region if the trap generated heat, 12.6% ± 36.5% longer if warm water was added to the trap, and 37.3% ± 46.0% longer if both cues were present (GLM, p > 0.03,  Fig. 6e, g). The spatial distribution of the remaining computed flight metrics did not vary if heat or warm water were added to the M-Tego (see Additional file 1: Fig. S10–S12).

## Discussion

The main hypothesis examined in this study was that due to a lack of the close-range host cues heat and humidity, mosquitoes do not approach counter-flow odour-baited traps closely enough, thus resulting in low capture efficiency. To systematically test this hypothesis, a novel trap was developed, the M-Tego, to provide these close-range host cues. First, the capture performance of this M-Tego with or without additional cues to that of the standing BG-Suna were compared in dual-choice tests and semi-field experiments. The flight behaviour of female mosquitoes around this M-Tego trap with or without additional heat and humidity were also compared.

It was found that, when the close-range host cues heat and humidity were present, more mosquitoes were lured to a region close to the rim of the trap tarpaulin bag. Mosquitoes were also observed to stay longer in this region where flight activity was high. This increase in mosquito attraction and time spent in this region when short-range host cues were present correlates with the concomitant increase in capture percentages.

These results are consistent with previous findings showing that heat and increased local humidity are important short-range host cues used by mosquitoes [9, 13, 17, 25]. Several odour-baited traps such as the HDT or CDC-type traps were shown to have increased capture rate when generating heat [17, 25]. Heat and increased local humidity have been classified as short-range cues because they cannot be detected from more than one metre, and work in synergy with host odour and CO_2_ to trigger landing behaviour [3, 9]. As such, it is not surprising that such cues are crucial for adhesive traps like the HDT because they only capture mosquitoes that land [17]. However, counter-flow traps capture mosquitoes while they fly, and therefore the function of short-range host cues for these traps was previously not clear. Then again, the higher flight activity observed near the M-Tego tarpaulin bag would suggest that landing on the bag occurred more often when these cues were present [17].

Similar behavioural changes were observed when either adding a heat source or warm water to the M-Tego. This occurred even though the heat source was placed on the top of the trap inlet while the warm water was inside the tarpaulin bag. This suggests that the circulating airflow allowed an approximately homogeneous mixing of the warmed air around the trap. This is confirmed by measurements (Additional file 1: Fig. S15) showing similar temperature increases in the full volume above the trap bag.

Results suggest that there is a saturation effect of adding heat and warm water on the change of flight activity near the trap (Fig. 6f), e.g., the benefit of adding both cues together is not the sum of their separate benefits. Moreover, despite including both sensory cues (heat and humidity), the benefit of adding warm water to the M-Tego was not clearly established because this addition of warm water was not found to significantly increase capture percentages of the M-Tego in laboratory conditions, but resulted in increased flight activity and time spent near the trap. A higher difference between the local humidity and ambient humidity might have resulted in significant difference in trapping performances. Thus, future research could investigate the effects of such differences between local and ambient values of temperature and relative humidity on capture performance, as well as how mosquito flight dynamic is affected by changes in the position of local heat and humidity sources.

To generate short-range host cues in the field, it is probably easier to use a heat source rather than warm water. The latter requires more labour as water needs to manually be warmed up, and evaporated water needs to be replaced. Also, small water bodies may attract mosquitoes to oviposit, and access to water needs to be carefully restricted. In addition, warming up water requires most likely more energy than directly using a heat source on the trap. Passive heating solutions may need to be considered to reduce energy consumption.

In all experiments, the heat source was powered using the same 12 V energy source used for the fan. The Nichrome heating wire used 9.6 W, and because the fan only used 3.4 W, adding the heating wire increased the power requirement of the trap by 282%. This power requirement may need to be lowered if the M-Tego is to be powered using solar panels in rural areas without a power grid.

Still, the M-Tego presents several advantages as a potential vector control tool against malaria. Like other counter-flow odour-baited traps, it uses artificial bait, and does not need to draw odours from living organisms such as humans or cattle. This greatly improves usability and repeatability, which are important when used as a monitoring tool. The M-Tego is easy to transport thanks to its small size, it has high modularity and can be folded like the BG-Sentinel, and its catch pot is easier to remove and empty than commonly used capture bags. Finally, despite having similar designs, the M-Tego without additional host cues captures between 2.4 to 2.9 as many *Anopheles* mosquitoes than the standing BG-Suna.

There was one striking difference in the flight dynamics of mosquitoes around the M-Tego when compared to the previously studied traps. Mosquitoes were found to elicit upward manoeuvres resulting in escapes above the inlet of the standing BG-Suna [18] and the BG-Sentinel [22]. Such manoeuvres were not observed above the M-Tego. This difference might help explain the superior capture percentages of M-Tego compared to the standing BG-Suna, even when no additional close-range host cues were present. Despite similarities between the two traps, the design of the M-Tego distinguished itself by its materials, its smaller size and the use of a transparent net through which the odour is released, instead of perforated hard plastic (see Additional file 1: Fig. S1). Therefore, the slightly different visual cues and airflow generated by the M-Tego may explain the lack of avoidance behaviour. The airflow speeds measured around the M-Tego were similar to the ones measured around the BG-Suna (see Additional file 1: Fig. S15 and [18]). However, it is possible that the fine net of the M-Tego resulted in a different turbulence level of the airflow, which might have changed how mosquitoes perceived the air movements [36]. The exact reason for this capture performance difference needs to be investigated further.

Except for the differences in flight behaviour near the trap inlet, the mosquito flight dynamics around the M-Tego were very similar to what has been observed around the standing BG-Suna and BG-Sentinel [18, 22]. However, because the cameras covered a larger volume than previous studies, it was possible to study the flight behaviour around the full trap and close to the back walls (see Additional file 1: Figs. S5, S7, S9 and S12). Similarly to what was observed for the standing BG-Suna and the BG-Sentinel, the flight activity was the highest close to the M-Tego, where mosquitoes had lower flight speed and higher angular speed [18, 22]. Such flight characteristics suggest that mosquitoes were host-seeking in this region [9]. Additionally, mosquitoes had high average flight speeds and accelerations in the region close to the inlet, and where the capture probability was high. Moreover, the size and shape of this capture region were similar to the ones of the capture region of the standing BG-Suna [18]. These similarities are not surprising considering that the two traps generate similar odour cues and airflow speeds (see Additional file 1: Fig. S15).

## Conclusions

By tracking mosquitoes in the vicinity of the newly developed M-Tego trap, their interaction with this odour-baited trap was described. Even though the role played by warm water is still ambiguous, adding a heat source to the trap resulted in large and significant increases in capture performance of the M-Tego in both laboratory and semi-field conditions. The results of this study suggest that these increases are due to a rise of flight activity close to the rim of the odour-release surface of the trap. The presence of the close-range host cues heat and increased humidity caused mosquitoes to approach the trap more often, and it retained mosquitoes for a longer period close to the trap. The combination of more approaches and increased retainment most likely explain the up to 129% increase in capture performance of the M-Tego with close-range host cues, compared to the M-Tego without such cues.

Additionally, in contrast to what was observed above the standing BG-Suna and BG-Sentinel [18, 22], it was shown that the average mosquito did not exhibit upward avoiding manoeuvres when flying above the M-Tego. This important difference could explain the 140% to 190% increase in capture performance of the M-Tego without additional host cues, relative to the standing BG-Suna.

This study showed that adding close-range host cues to odour-baited traps can greatly increase their capture performance. This knowledge may help the development or improvement of vector control tools and strategies, especially for malaria vectors. In addition, this study showed that the M-Tego has the potential to be valuable for the monitoring and control of *Anopheles* mosquitoes.

## Supplementary information

**Additional file 1:** Supplementary figures S1–S15, and legends of the supplementary datasets S1–S3 and the table S1.

**Additional file 2:** Supplementary datasets S1–S3 and the table S1.

## Data Availability

The datasets generated and analysed during the current study are available in the following Dryad repository: 10.5061/dryad.k6djh9w47.

## Abbreviations

GLM: generalized linear model
AIC: Akaike information criterion
std: standard deviation
P_i_: positional likelihood of mosquitoes in a cell *i*
N: sum of the length of all tracks in the filmed volume (i.e. the total number of frames)
n_i_: sum of the length of the parts of these tracks with mosquitoes flying in the cell *i*
I: total number of cells within the filmed volume
r_bag_: radius of the trap bag
r_inlet_: radius of the inlet of the trap
