# Supplementary material for: Lure, retain, and catch malaria mosquitoes. How heat and humidity improve odour-baited trap performance
Source: Malar J. 2020 Oct 7;19:357. doi: 10.1186/s12936-020-03403-5 (PMC7542916; doi:10.1186/s12936-020-03403-5)
Supplement: Supplementary file 1 — Additional file 1: Supplementary figures S1–S15, and legends of the supplementary datasets S1–S3 and the table S1. [file 12936_2020_3403_MOESM1_ESM.pdf]

Electronic Supplementary Material for

Lure, retain, and catch malaria mosquitoes. How heat and humidity improve odour-baited trap performance

Antoine Cribellier[1], Jeroen Spitzen[2], Henry Fairbairn[1,3], Cedric van de Geer[1,2,3,4], Johan L. van Leeuwen[1], Florian T. Muijres[1]

[1]Experimental Zoology Group, Wageningen University, Wageningen, The Netherlands
[2]Laboratory of Entomology, Wageningen University, Wageningen, The Netherlands.
[3]Faculty of Industrial Design Engineering, Delft University of Technology, Delft, The Netherlands.
[4]Ifakara Health Institute, Ifakara, Tanzania.

[*]corresponding author: florian.muijres@wur.nl

Consisting of:

- Legends of the databases S1, S2, and S3 and of the table S1
- Supplementary figures S1-S15

Legends of the supplementary datasets and table

Database S1: Experimental conditions and results from the dual-choice testing and the mosquito flight tracking in the laboratory (Wageningen, the Netherlands).

Database S2: Experimental conditions and results from the semi-field experiments (Ifakara, Tanzania).

Database S3: Mosquito flight tracks around the M-Tego with or without additional host cues and experimental metadata. Matlab .mat file with the three-dimensional tracks of the flying mosquitoes were obtained as described in the materials and methods. Flight tracks were described as the {x, y, z} coordinates in meters of the mosquito at each video frame. Coordinates are in the world reference frame as defined in figure 1, with z oriented vertically up, and its origin at the centre of the trap inlet.

Table S1: Table with generalized linear models (GLM) results and model selection criterions used to select the models presented in the manuscript.

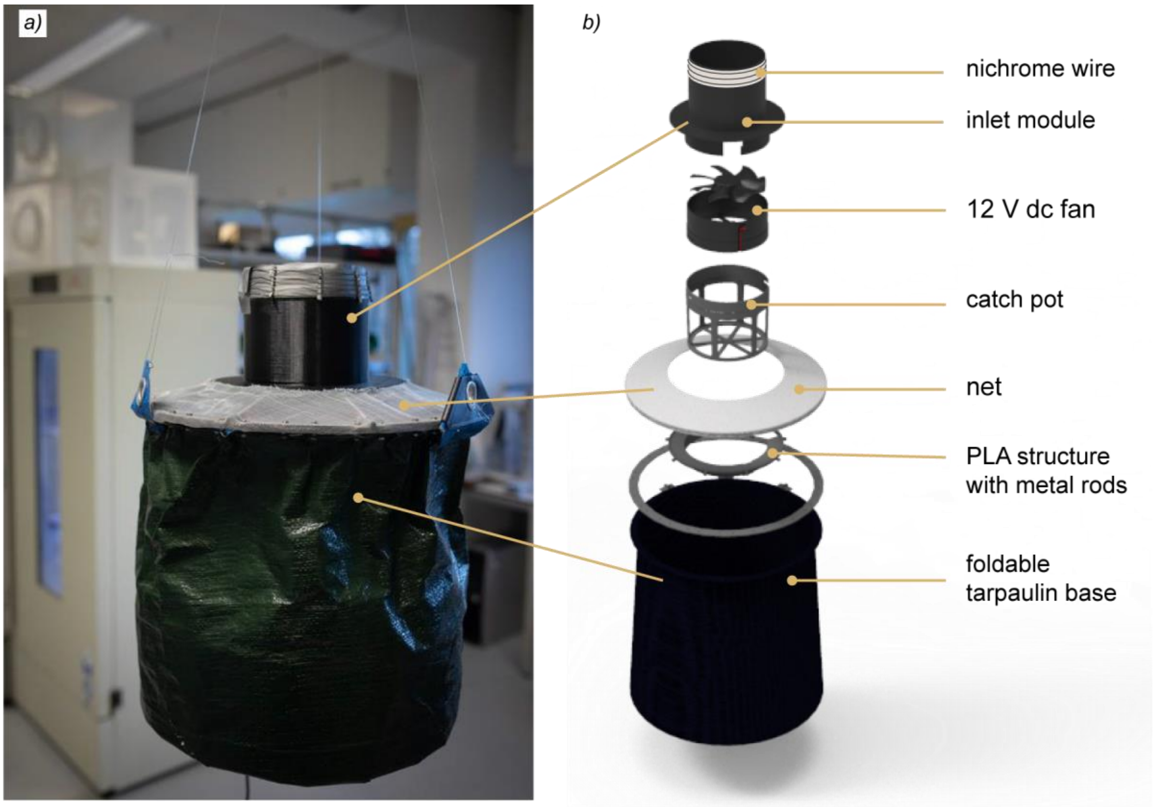

Supplementary figure 1. Pictures and exploded view of the M-Tego prototype mosquito trap. (a) Picture of the M-Tego trap used during the experiments (Photo by Sven Menshel). (b) Exploded view of the trap showing its various parts.

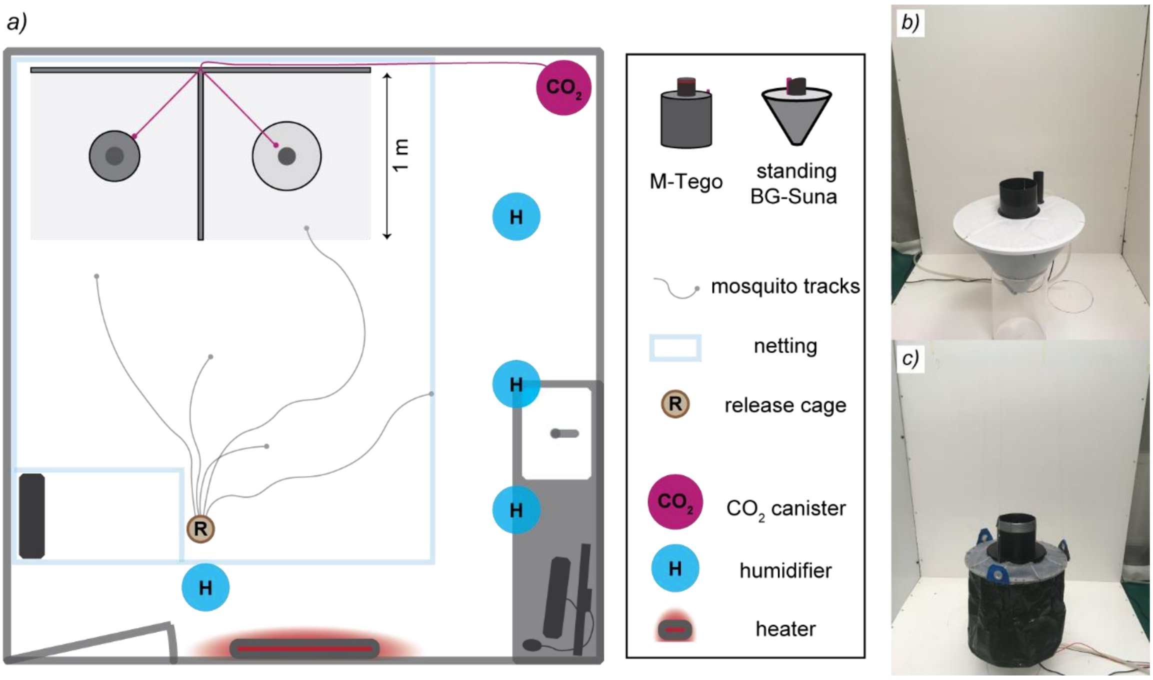

Supplementary figure 2. Experimental setup used for the dual-choice experiments. (a) Top-down view of the room used for the laboratory experiments at Wageningen University (Wageningen, The Netherlands). Two traps (standing BG-Suna or M-Tego) were placed on the left and right of the dual-choice setup (alternated between replicates). The traps were separated by a vertical white wall. A MB5 blend was placed inside each of the traps and both were connected to the CO_2 canister. Several humidifiers and one heater were regulating the relative humidity and temperature in the room. (b) BG-Suna placed in the left side of the setup. (c) M-Tego placed in the right side of the setup.

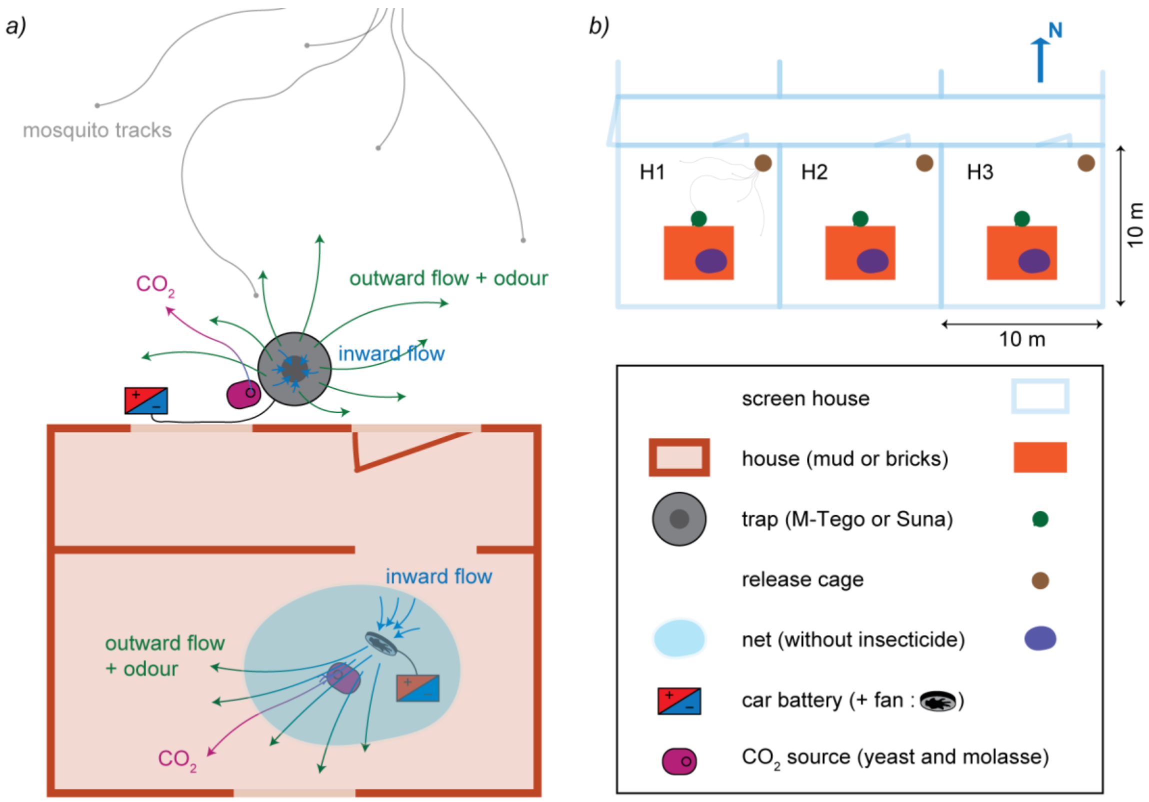

Supplementary figure 3. Experimental setup of the semi-field experiments. (a) Top-down view of the setup inside one screen house at Mosquito City in Ifakara (Tanzania). A standing BG-Suna or M-Tego was placed outside the house. Inside the bed net in the house, we placed a fan with an MB5 blend and a molasses-based CO_2 source to mimic human presence. (b) Top-down view of all three screen houses used in the semi-field experiments.

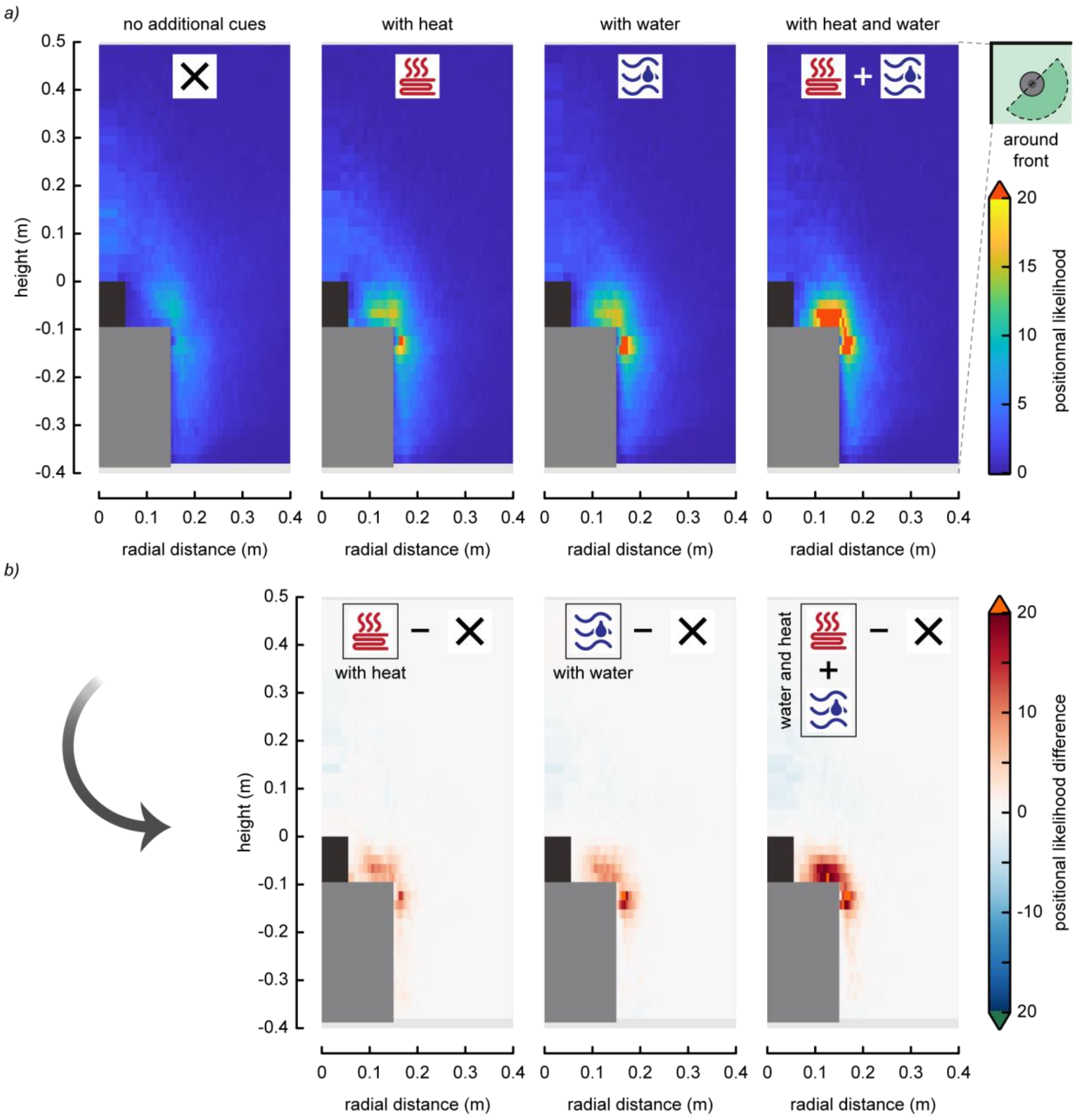

Supplementary figure 4. Spatial distribution of the positional likelihood of mosquito flying around the M-Tego with or without additional cues (half the volume, as indicated in the top right of a). (a) Radial - vertical heat maps of positional likelihood around the M-Tego with or without additional host cues. As indicated in the top right of a, all heat maps have been computed on only half the volume to avoid overlapping with blind spots behind the trap. (b) Difference between the heat maps of positional likelihood around the M-Tego with additional host cues (heat and/or water) and the heat maps of positional likelihood around the M-Tego without additional host cues. Average cell size = 19 x 3 mm.

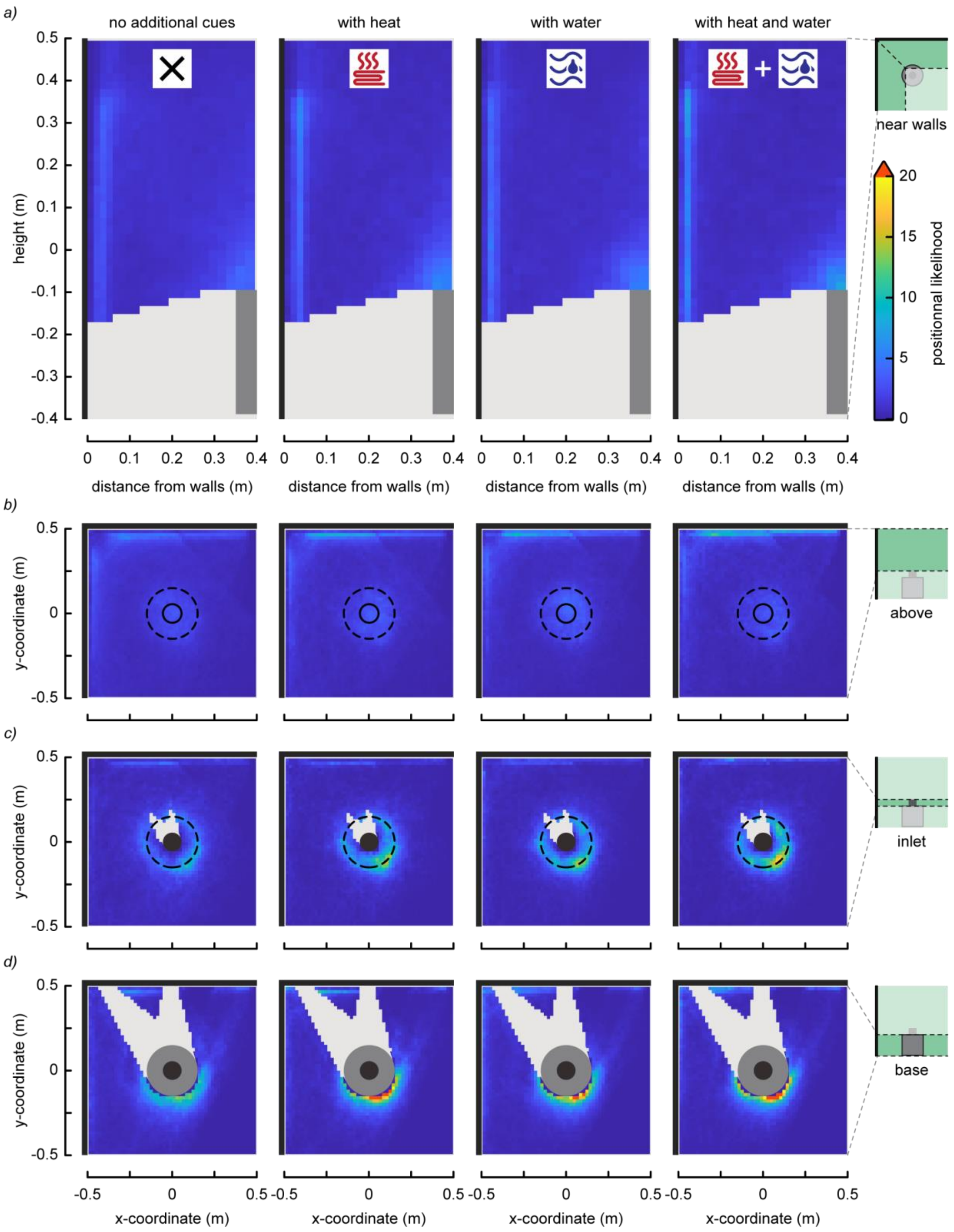

Supplementary figure 5. Positional likelihood of mosquito flying within various volumes around the M-Tego with or without additional cues (volumes around the trap are indicated on the right). (a) Vertical heat maps of positional likelihood close to the walls behind the various conditions, as indicated on the top right. (b,c,d) Top-down view of the positional likelihood above the trap inlet (b), at the inlet height (c) and around the trap base (d). Average cell size = 19 x 19 mm.

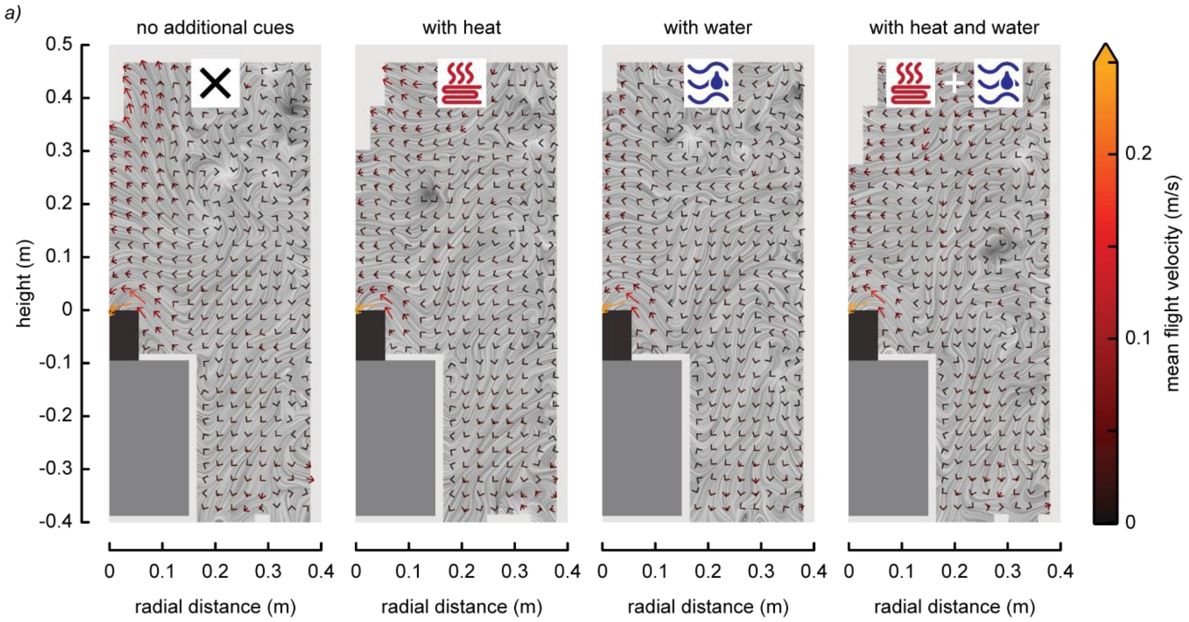

Supplementary figure 6. Average velocity fields and streamlines of mosquitoes flying around the M-Tego with or without additional host cues (cues as indicated by the symbols). Vectors show the average velocity fields in the radial and vertical direction each cell of average size 27.5 x 27.5 mm. Velocity vectors resulting from fewer than 20 tracks were discarded. Streamlines were computed using LIC (Line Integral Convolution).

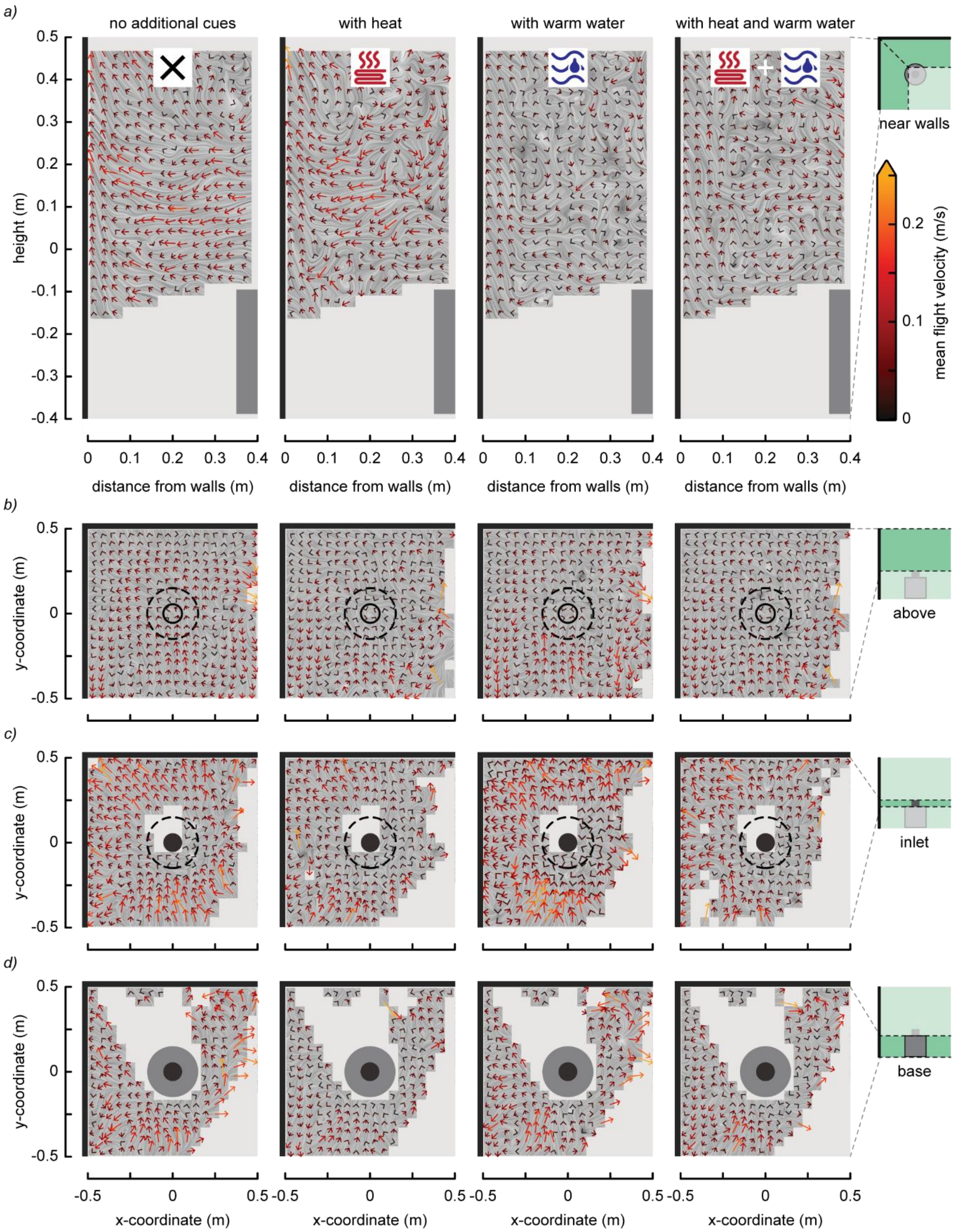

Supplementary figure 7. Average velocity fields and streamlines of mosquitoes flying within various volumes around the M-Tego with or without additional cues (volumes around the trap are indicated on the right in dark green). (a) Vertical view of the average velocity fields close to the walls. Each vector consists of the average velocity in that cell of average size 27.5 x 27.5 mm. All velocity vectors resulting from less than 20 tracks were discarded. Streamlines were computed using LIC (Line Integral Convolution). (b,c,d) Top-down velocity fields above the trap inlet (b), at the inlet height (c) and around the trap base (d). Cell size is 55 x 55 mm.

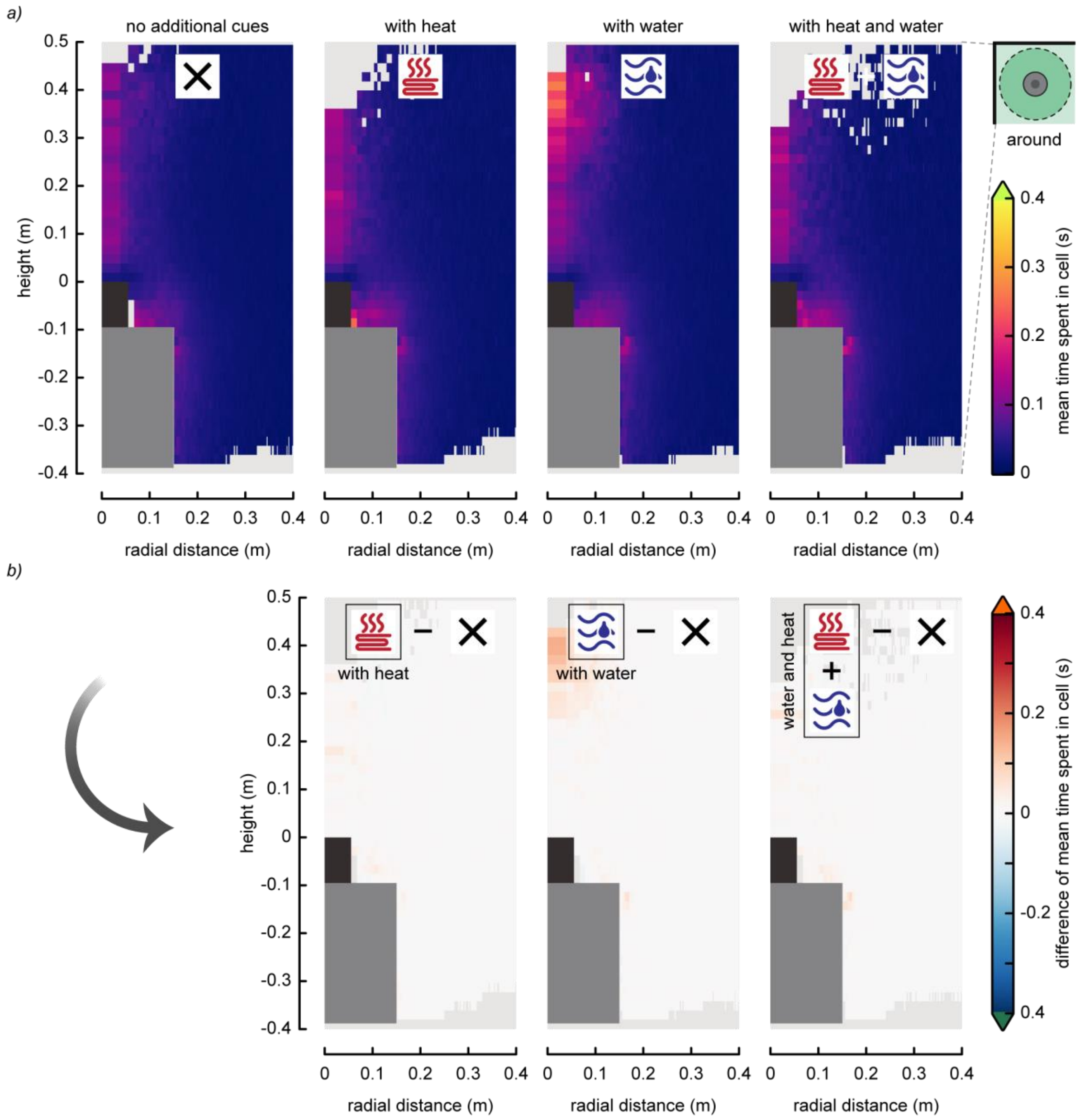

Supplementary figure 8. Spatial distribution of time spent by mosquitoes around the M-Tego with or without additional cues. (a) Radial - vertical heat maps of the average time spend by mosquitoes around the M-Tego with or without additional host cues. (b) Difference between the heat maps of time spend around the M-Tego with additional host cues (heat and/or water) and the heat maps of time spend around the trap without additional host cues. Average cell size = 19 x 3 mm.

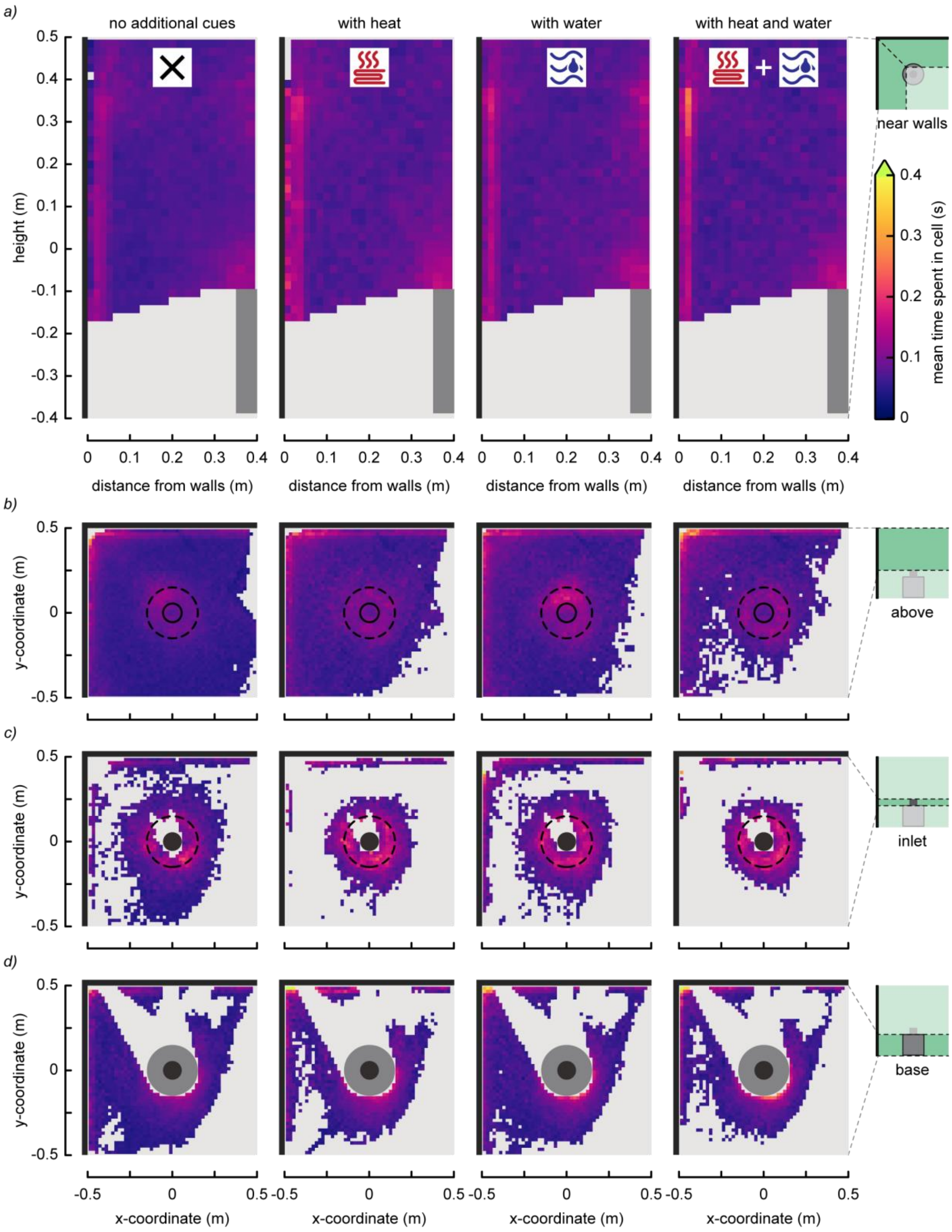

Supplementary figure 9. Spatial distribution of time spent by mosquitoes within various volumes around the M-Tego with or without additional cues (volumes around the trap are indicated on the right in dark green). (a) Vertical heat maps of the average time spend by mosquitoes close to the walls behind the traps. (b,c,d) Top-down view of the average time spend by mosquitoes above the trap inlet (b), at the inlet height (c), and around the trap base (d). Average cell size = 19 x 19 mm.

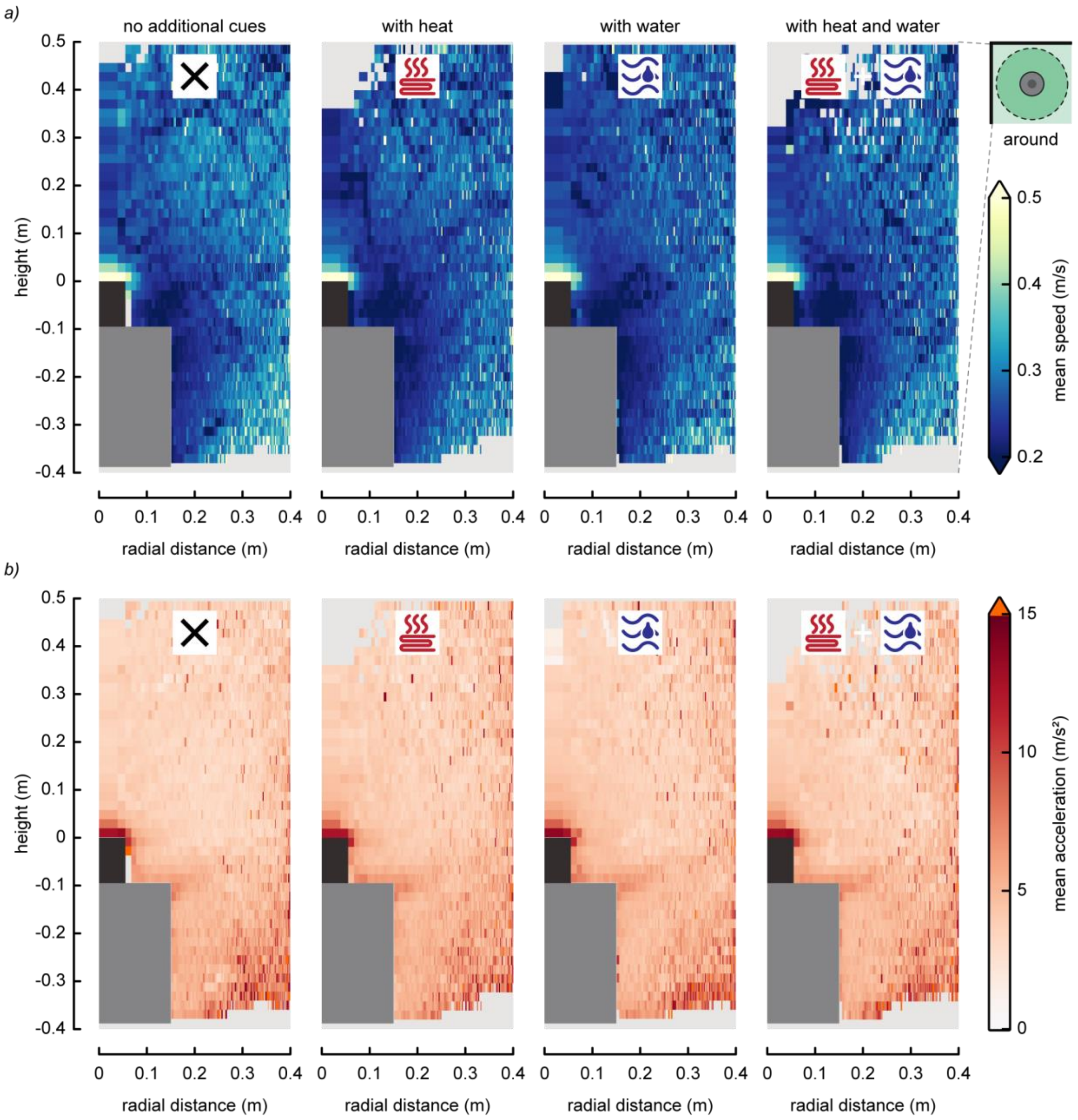

Supplementary figure 10. Spatial distribution of flight speed and vertical acceleration of mosquitoes flying around the M-Tego with or without additional cues. (a) Radial - vertical heat maps of the average flight speed around the M-Tego with or without additional cues. (b) Radial - vertical heat maps of the average acceleration of mosquitoes flying around the M-Tego with or without additional cues. Average cell size = 19 x 3 mm.

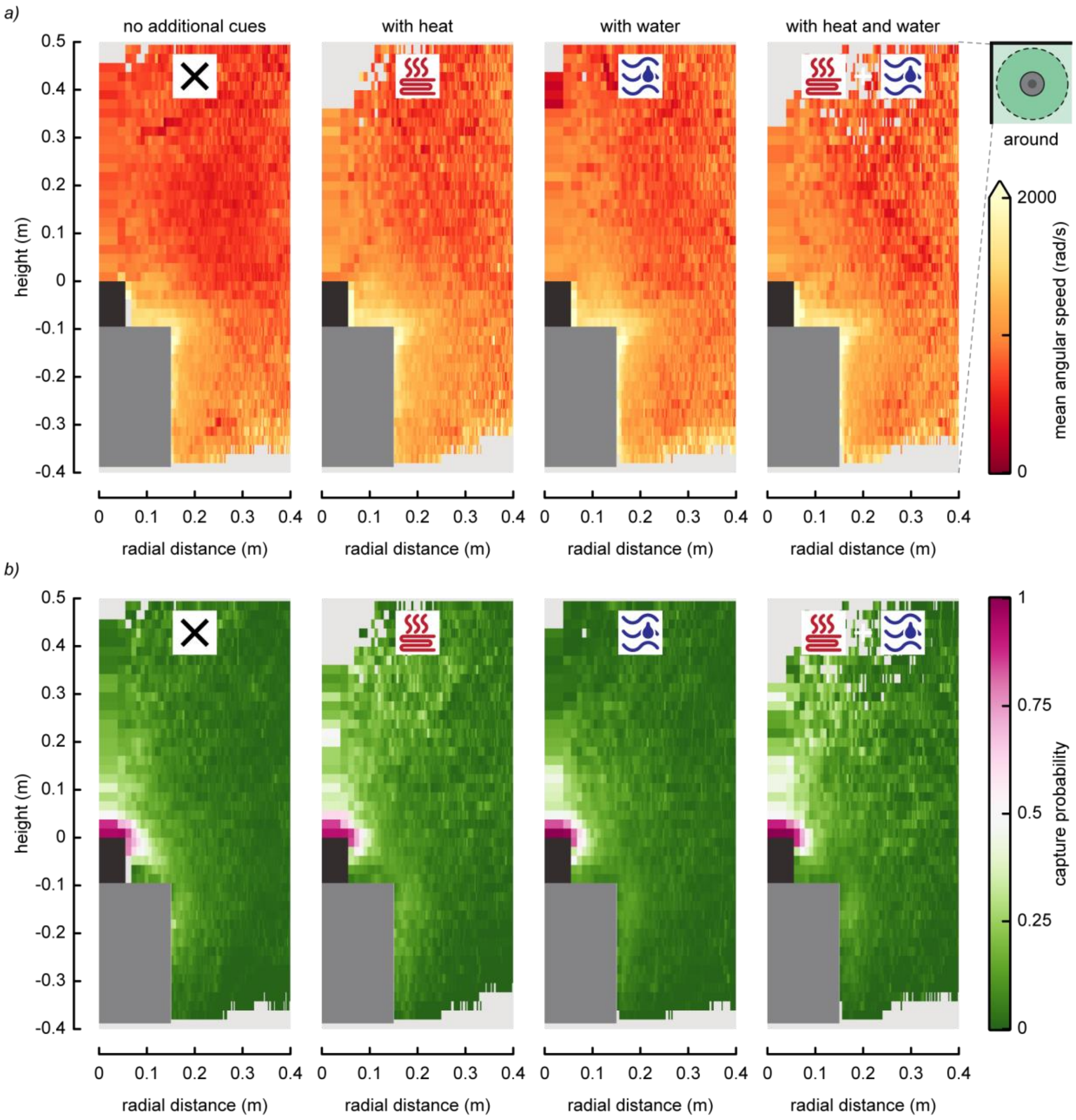

Supplementary figure 11. Spatial distribution of angular flight speed and capture probability of mosquitoes flying around the M-Tego with or without additional cues. (a) Radial - vertical heat maps of the average angular speed of mosquitoes. (b) Radial - vertical heat maps of mosquitoes capture probability around the M-Tego with or without additional cues. Average cell size = 19 x 3 mm.

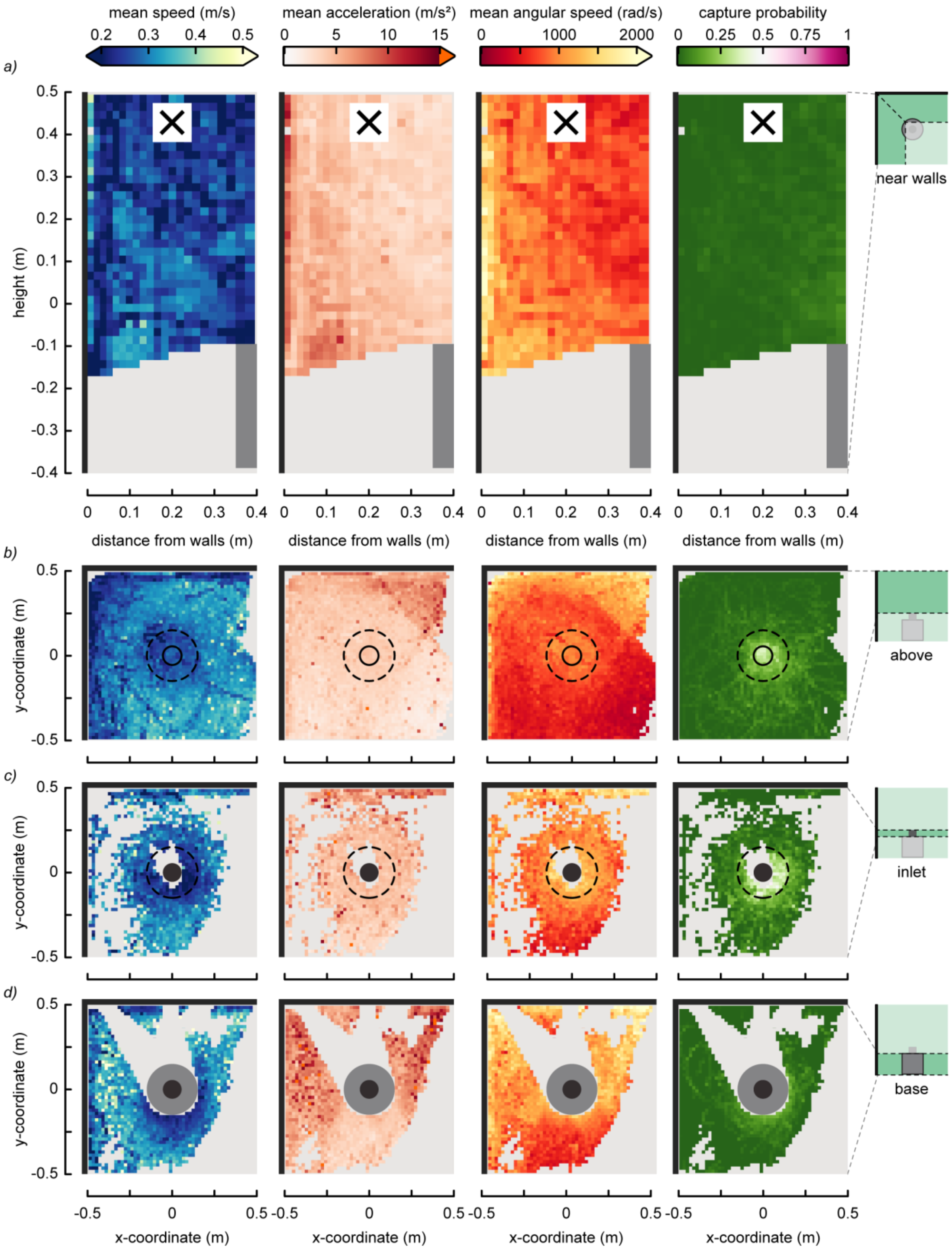

Supplementary figure 12. Spatial distribution of various flight dynamics metrics of mosquitoes flying within various volumes around the M-Tego without additional cues (volumes around the trap are indicated on the right in dark green). (a) Vertical heat maps of average flight speed, average acceleration, average angular speed and capture probability near the wall. (b,c,d) Top-down view of these metrics above the trap inlet (b), at the inlet height (c) and around the trap base (d). Average cell size = 19 x 19 mm.

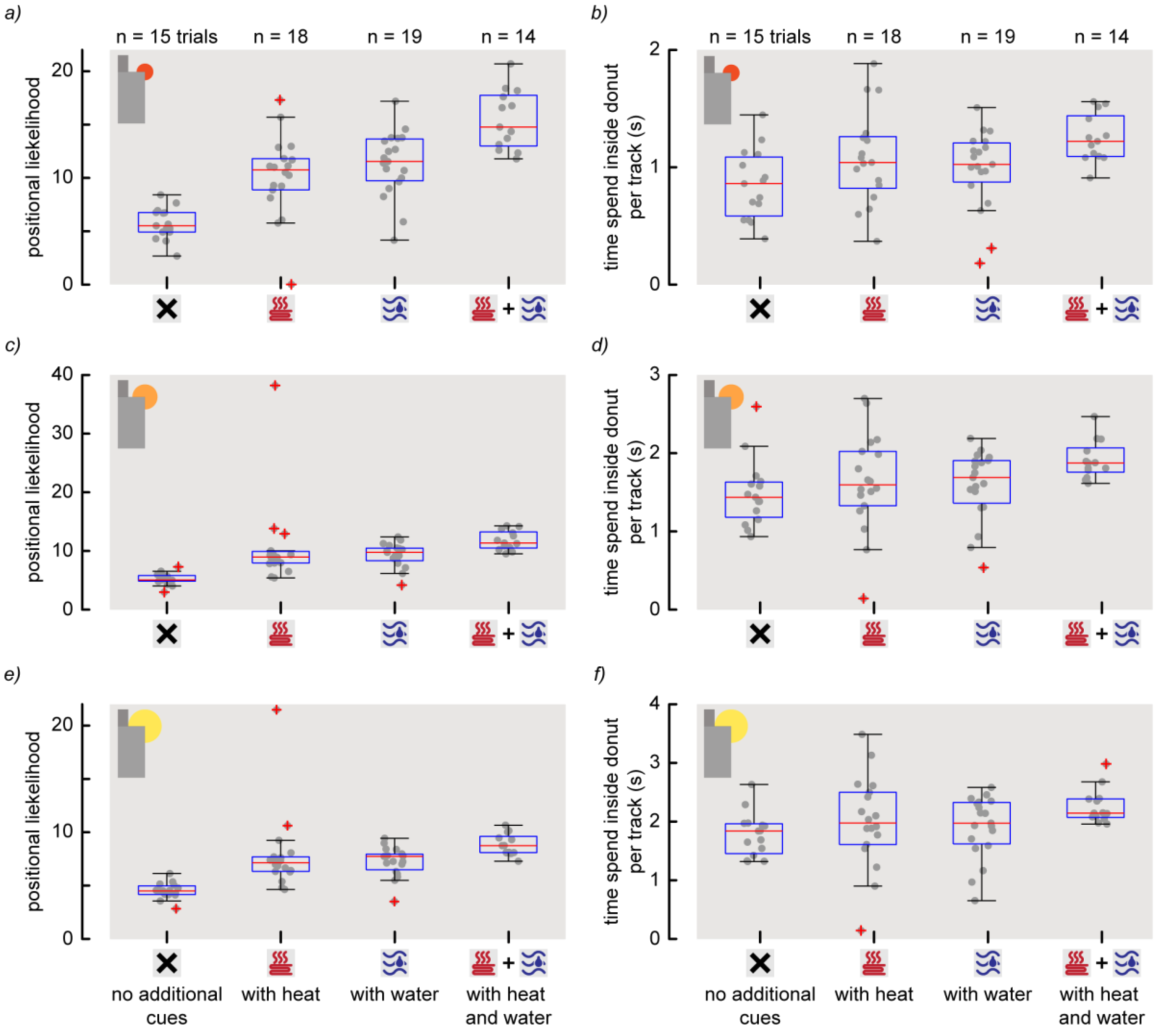

Supplementary figure 13. Positional likelihood and time spent by mosquitoes in the donut-shaped region close to the trap edge. (a,c,e) Box plots of the percentage of points detected inside three volumes of various sizes over the total number of detected points for each trial and in function of if the trap was with or without additional cues. (b,d,f) Box plots of the average time spend by mosquitoes inside the three donuts of various sizes for each trial and in function of if the trap was with or without additional cues. The number of tracks used for computing both metrics were 7153 (a,b), 9011 (c,d) and 10565 (e,f).

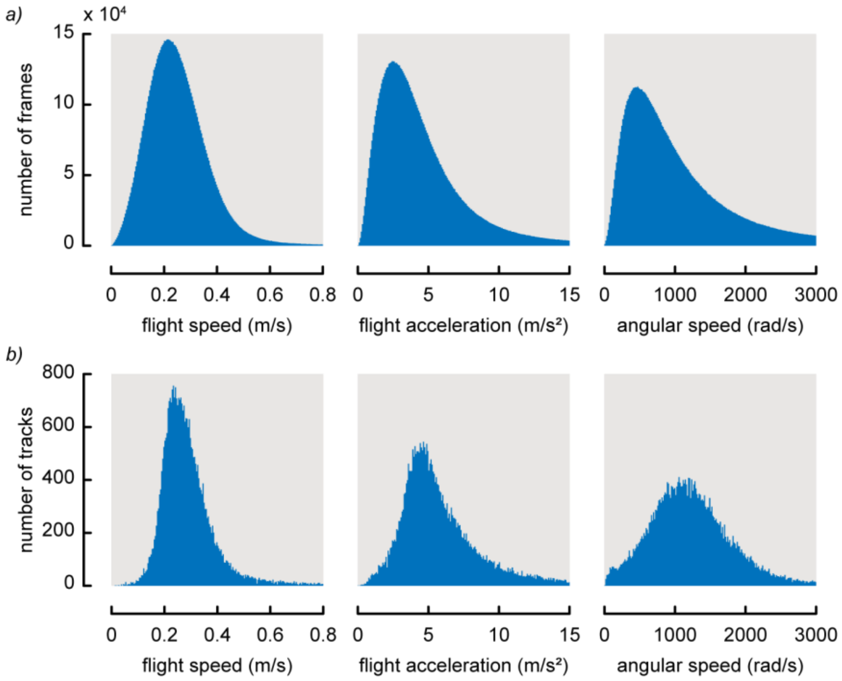

Supplementary figure 14. Histograms of several flight metrics around the M-Tego. Histograms of mosquito flight speed, acceleration and angular speed for all recorded frames (a) or averaged per tracks (b).

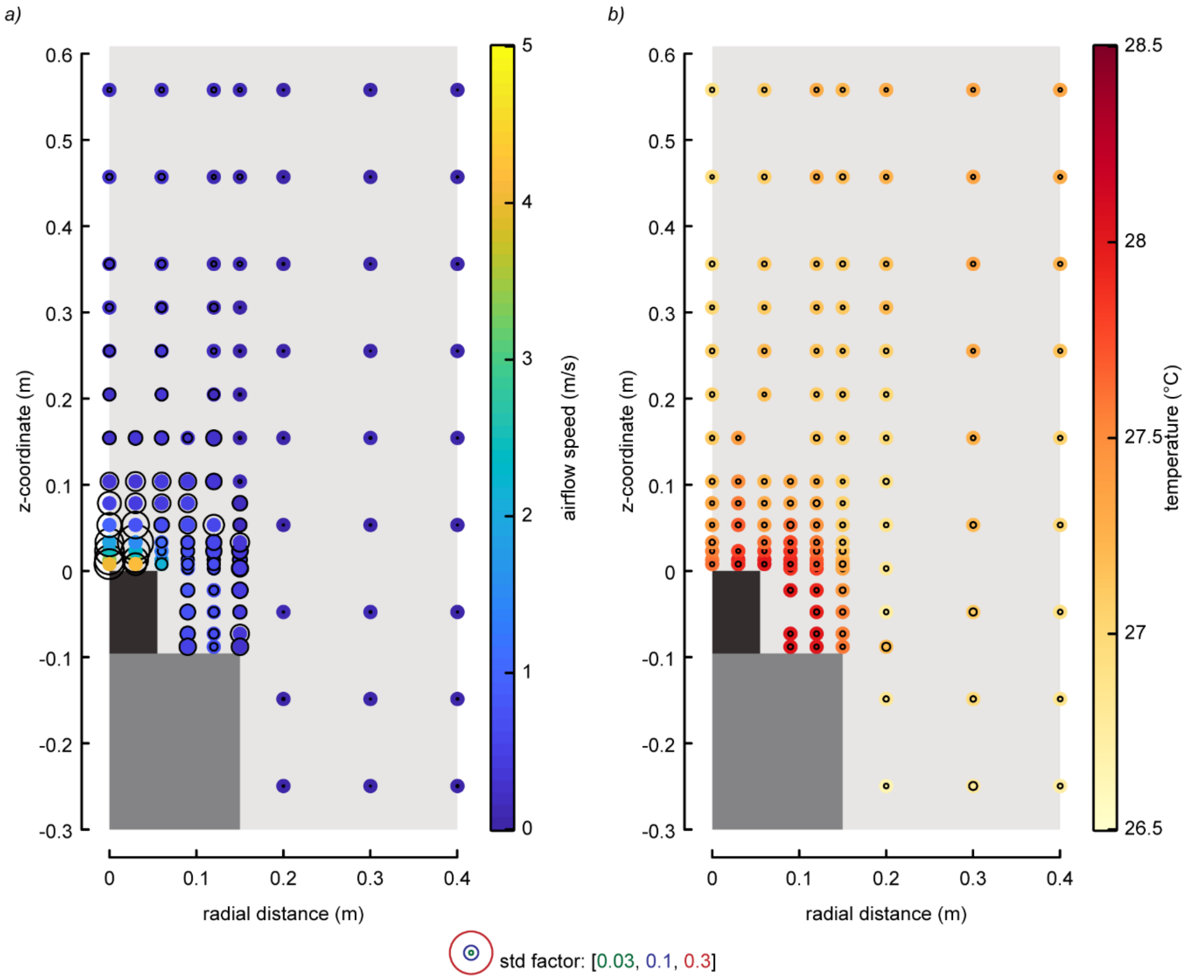

Supplementary figure 15. Airflow and temperature measurements around the M-Tego. (a) airspeeds generated by the M-Tego at various position around the trap. These airspeeds have been computed from the vertical and radial airspeeds measured with a hotwire anemometer (tetso 405i). These are similar to the ones measured around the BG-Suna (Cribellier et al., 2018). (b) Temperature generated by the nichrome wire on the top of the M-Tego inlet (with fan turned on). These temperatures have been measured at various positions around the trap. Standard deviations are shown using circles of various sizes (the legend is showing corresponding factors).
